# *White Leaf and Panicle 2*, encoding a PEP-associated protein, is required for chloroplast biogenesis under heat stress in rice

**DOI:** 10.1093/jxb/erx332

**Published:** 2017-10-16

**Authors:** Yusong Lv, Gaoneng Shao, Jiehua Qiu, Guiai Jiao, Zhonghua Sheng, Lihong Xie, Yawen Wu, Shaoqing Tang, Xiangjin Wei, Peisong Hu

**Affiliations:** 1 State Key Laboratory of Rice Biology, China National Rice Research Institute, Hangzhou, China; 2 National Key Laboratory of Crop Genetic Improvement, Huazhong Agricultural University, Wuhan, China

**Keywords:** chloroplast biogenesis, heat stress, PEP-associated protein, redox balance, rice, *White Leaf and Panicle 2*, *WLP2*

## Abstract

The plastid-encoded RNA polymerase (PEP) plays an important role in the transcription machinery of mature chloroplasts, yet details of its function remain elusive in rice. Here, we identified a novel PEP-associated protein (PAP), WLP2, based on its two allelic white leaf and panicle mutants, *wlp2s* and *wlp2w*. The two mutants were albino lethal at high temperatures and showed decreased chlorophyll accumulation, abnormal chloroplast ultrastructure, and attenuated photosynthetic activity. Map-based cloning suggested that *WLP2* encodes a putative pfkB-type carbohydrate kinase family protein, which is homologous to fructokinase-like 1 (AtFLN1) in Arabidopsis. *WLP2* is mainly expressed in green tissues and its protein localizes in chloroplasts. Expression levels of PEP-encoded genes, chloroplast development genes and photosynthesis-related genes were compromised in *wlp2* mutants, indicating that *WLP2* is essential for normal chloroplast biogenesis. Moreover, WLP2 and its paralog OsFLN2 can physically interact with thioredoxin OsTRXz to form a TRX-FLN regulatory module, which not only regulates transcription of the PEP-encoded genes but also maintains the redox balance in chloroplasts under heat stress. Furthermore, the *wlp2w* mutant gene represents a potential advantage in enhancing seed purity and high-throughput breeding. Our results strongly indicate that WLP2 protects chloroplast development from heat stress via a TRX-FLN regulatory module in rice.

## Teaser

*WLP2*, encoding a PEP-associated protein, and its paralog OsFLN2 can interact with OsTRXz to form a TRX-FLN regulatory module to protect chloroplast development from heat stress in rice.

## Introduction

Chloroplasts are specific organelles in higher plants that are responsible for photosynthesis and other important metabolic pathways. As a semi-autonomous organelle, each chloroplast contains ~100–250 genes, which encode proteins related to photosynthesis or the plastid gene transcription machinery (reviewed in Wicke *et al.*, 2011). Plastid genes in angiosperms are mainly transcribed by two types of RNA polymerases: the nuclear-encoded RNA polymerase (NEP) and the plastid-encoded RNA polymerase (PEP) (Börner *et al.*, 2015; Shiina *et al.*, 2005). NEP is predominantly responsible for the accurate transcription of plastid housekeeping genes, including PEP core subunits and genes involved in basic plastid functions. In contrast, PEP drives the mass production of photosynthetic gene transcripts necessary for generating fully active chloroplasts and is responsible for over 80% of all primary plastid transcripts in mature chloroplasts (Puthiyaveetil *et al.*, 2010; Zhelyazkova *et al.*, 2012). The PEP complex is composed of four plastid-encoded core subunits (rpoA, rpoB, rpoC1, and rpoC2) and several nuclear-encoded proteins, such as sigma factors and polymerase-associated proteins (PAPs). The rpo subunits generate the core complex, which is coated by additional proteins such as PAPs to build a larger chloroplast-specific PEP complex (Belbin *et al.*, 2016; Pfannschmidt *et al.*, 2015; Steiner *et al.*, 2011).

Previous investigations suggested that PAPs, which display potential functions such as DNA/RNA metabolism, PEP complex protection, and redox regulation, can affect the activity and integrity of the PEP complex to further regulate the chloroplast transcriptional machinery (Pfalz *et al.*, 2016; Pfannschmidt *et al.*, 2015). Studies on knockout mutants of PAP genes with white/ivory leaves, lower or missing PEP activity, and higher NEP activity have supported the notion that PAPs are indispensable for PEP-mediated transcription. Recently, at least 12 different PAPs have been identified in Arabidopsis (Pfalz *et al.*, 2006, 2016; Steiner *et al.*, 2011). PAP1, which contains a DNA-binding SAP domain, was first reported as a chloroplast nucleoid-localized transcription factor in the PEP complex that mediates DNA/RNA binding (Yagi *et al.*, 2012). PAP4 (FSD2) and PAP9 (FSD3) are two superoxide dismutases found in the PEP complex, which may form a heteromeric protein complex scavenging against oxidative stress to protect the PEP complex during early chloroplast development (Myouga *et al.*, 2008). Furthermore, some regulatory proteins of PAPs can fine-tune the transcription of PEP-mediated genes. PAP6 (FLN1) and its paralogous protein FLN2, two pfkB-type carbohydrate kinase family members, are involved in the regulation of PEP-dependent gene transcription in chloroplasts (Arsova *et al.*, 2010, Gilkerson *et al.*, 2012). FLN1 can interact with DELAYED GREENING 238 (DG238), which is involved in regulating chloroplast development and PEP-dependent gene expression (Wang *et al.*, 2016*b*). PAP10 (TRXz), encoding a thioredoxin family protein, has been confirmed to physically interact with PAP6 and FLN2 and regulate PEP activity, possibly via redox regulation of targeted proteins (e.g. PAP6 and FLN2) during chloroplast biogenesis (Arsova *et al.*, 2010; Wimmelbacher and Börnke, 2014). Moreover, proteins such as PAP3 (pTAC10), PAP5 (pTAC12), PAP8 (pTAC6), and PAP12 (pTAC7) with unknown functions have been suggested to be involved in PEP-mediated plastid gene expression and to promote chloroplast development (Chang *et al*., 2017; Kindgren and Strand, 2015; Pfalz *et al.*, 2006). Although there has been rapid progress in the understanding of the PEP complex in Arabidopsis, studies concerning the transcription process in chloroplasts, and especially the functions of PEP the complex, are notably limited in important crops such as rice. In particular, WSL3, a homologous protein of Arabidopsis PAP1, has been identified to be essential for early chloroplast development in rice (Wang *et al.*, 2016*a*). Thus, it is of great significance to unravel the precise functions of proteins in the PEP complex in rice, as it will contribute to better understanding of chloroplast biogenesis and the highly efficient photosynthesis system in rice.

Here, we identified a novel PAP (WLP2) in rice by a map-based cloning strategy. *wlp2* mutants exhibited albino lethality with elevated reactive oxygen species (ROS) production at high temperatures. Transcription levels of PEP-mediated genes during chloroplast biogenesis were significantly decreased, implying that WLP2 is involved in the regulation of PEP-mediated gene transcription and the heat stress response during chloroplast development in rice.

## Materials and methods

### Plant materials and growth conditions

*wlp2*s and *wlp2w* mutants were identified from an ethyl methanesulfonate (EMS)-induced population derived from *japonica* rice cv. Zhonghua11. An F_2_ population derived from the cross between *wlp2s* and *indica* cv. Nanjing11 was used for gene mapping. All plants were grown in the Hangzhou paddy fields during summer. The wild-type and mutant plants were also grown in a growth chamber (12/12 h light/dark; light intensity 300 μmol m^–2^ s^–1^) at a constant temperature of either 32, 28, or 22 °C.

### Chlorophyll content, transmission electron microscopy, photosynthetic rate, and chlorophyll fluorescence analysis

Leaf chlorophyll content was determined according to the method described by Wu *et al.* (2007). Chloroplast structures in the third leaf of both mutants and wild-type plants were examined using transmission electron microscopy (TEM) as described elsewhere (Inada *et al.*, 1998) with minor modifications. Samples were viewed with a Hitachi H-7650 transmission electron microscope. The LI-6400 portable photosynthetic system (LI-COR Biosciences) was used to detect the net photosynthetic rate in leaves. Chlorophyll fluorescence analyses were performed with a PAM-2000 portable chlorophyll fluorometer (Walz).

### Mapping and cloning of *WLP*2

To fine map the *WLP2* locus, 2348 individuals with the mutant phenotype were selected from the F_2_ population, and 16 new simple sequence repeat, InDel markers were developed (see Supplementary Table S1 at JXB online). The full-length coding region of the candidate gene of *WLP2* was isolated from cv. Zhonghua11 by PCR (Supplementary Table S1), subcloned into the binary vector pCAMBIA1305-GFP under the control of the rice *Ubiqutin1* promoter, and then introduced into the two mutants through *Agrobacterium*-mediated transformation.

### RNA extraction, qRT-PCR, and RNA-seq analysis

Total RNA was extracted using Trizol reagent (Invitrogen) following the manufacturer’s procedures. First-strand cDNA was reverse-transcribed by using oligo(dT) as primer. The transcription of selected genes was assessed using quantitative real-time PCR (qRT-PCR) and choosing the rice *ubiquitin* gene (GenBank accession number AF184280) as internal reference (primers are listed in Supplementary Table S1). RNA-sequencing (RNA-seq) analysis was performed on an Illumina Hiseq2000/2500 (LC Sciences) following the vendor’s recommended protocol and single end sequencing was performed on an Illumina Hiseq2500 instrument (LC Sciences). Significantly differentially expressed genes were identified, considering a *P*-value ≤0.05 and a log_2_ fold-change (log_2__FC) ≥1 as significant. Functional analysis of these genes was carried out by using the Gene Ontology (GO) analysis tool (http://www.geneontology.org/). The RNA-seq data have been submitted to the Gene Expression Omnibus (GEO) database (accession number GSE98885).

### Histochemical analysis

GUS histochemical assays were performed as described previously (Hull and Devic, 1995). The formation of hydrogen peroxide was detected by 3,3ʹ-diaminobenzidine (DAB) staining as described previously (Thordal-Christensen *et al.*, 1997). To detect superoxide anion radicals, rice leaves were exposed to nitroblue tetrazolium (NBT) staining as described previously (Thordal-Christensen *et al.*, 1997). For the 2ʹ,7ʹ-dichlorofluorescein diacetate (H_2_DCFDA) experiment, the protocol was adapted from Leshem *et al.* (2006). Chlorophyll autofluorescence and oxidized H_2_DCFDA were visualized using an Olympus FV300 confocal microscope.

### Subcellular localization of WLP2

The coding sequence of *WLP2* and six truncated sequences of *WLP2* were amplified by PCR. The PCR products were cloned into the pAN580-GFP vector and then transformed into rice protoplasts according to the protocols described previously by Zhang *et al.* (2011).

### Yeast two-hybrid and bimolecular fluorescence complementation assays

The coding region of OsTRXz was cloned into the bait vector pGBKT7, while WLP2 and OsFLN2 were cloned into the prey vector pGADT7. A yeast two-hybrid assay was performed following the manufacturer’s instructions (Clontech). Full-length OsTRXz, OsFLN2, and WLP2 were amplified by PCR and inserted into the binary vectors pSPYCE and pSPYNE (Waadt *et al.*, 2008). For enhanced yellow fluorescent protein visualization, the excitation wavelength was 488 nm and emission was captured at a wavelength of 527 nm. The primers used for vector construction are described in Supplementary Table S1.

### *In vitro* pull-down assay and western blots

WLP2, OsFLN2, and OsTRXz were inserted into the pFast-bac1, pET28a, and pGEX4T-1 vectors to generate the plasmids His-WLP2, His-OsFLN2, and GST-OsTRXz, respectively (primer sequences are listed in Supplementary Table S1). Proteins including fusions and empty tags were expressed in *Escherichia coli* BL21 cells and then purified. GST or GST-OsTRXz coupled beads were used to capture His-WLP2 or His-OsFLN2. The pull-down analyses were performed as described by Zhang *et al.* (2015).

For immunoblot analyses, total proteins were isolated from the leaves of wild-type, *wlp2s*, *wlp2w*, and OE-16 seedlings. The proteins were separated by sodium dodecyl sulfate-polyacrylamide gel electrophoresis, transferred to polyvinylidene difluoride membranes, immunoblotted with corresponding antibodies, and detected using High-sig ECL Western Blotting Substrate (Tanon).

## Results

### *wlp2* mutants exhibit white leaf and panicle phenotypes under high-temperature conditions

**Fig. 1.**
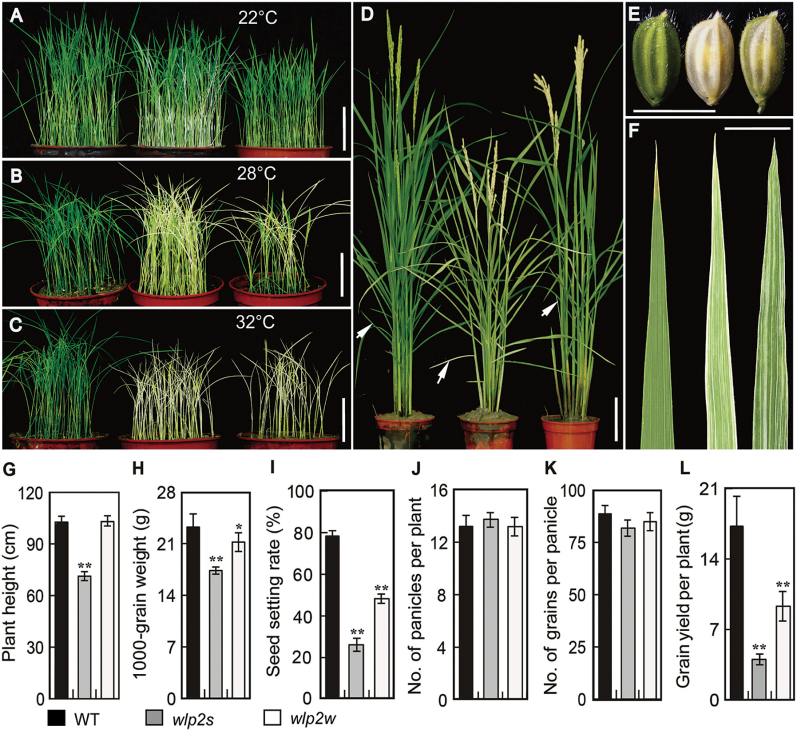
Phenotypic characterization of the *wlp2* mutants. (A–D) Three-week-old seedlings of wild-type (WT), *wlp2s*, and *wlp2w* (from left to right) grown at 22 °C (A), 28 °C (B), and 32 °C (C),. Bars=5 cm. (D) WT*, wlp2s*, and *wlp2w* plants (from left to right) at the heading stage grown in a paddy field in the summer of 2013 (mean temperature at the heading stage ~34.5 ºC). Bar=10 cm. (E) Spikelets and (F) basal leaves (white arrows in D) of WT, *wlp2s*, and *wlp2w*. Bars=0.5 cm. (G–L) Plant height (G), weight of 1000 grains (H), seed setting rate (I), number of panicles per plant (J), number of grains per panicle (K), and grain yield per plant (L) of WT, *wlp2s*, and *wlp2w*. Data in G–L are shown as means±SD from five individual replicates. Asterisks indicate statistical significance between WT and mutants, as determined by Student’s *t*-test: *P<0.05, **P<0.01.

The two mutants (*wlp2s* and *wlp2w*) displaying white leaf and panicle phenotypes were derived from EMS-treated *japonica* rice cv. Zhonghua11. An allelism test showed that the white leaf and panicle phenotypes of the mutants *wlp2s* and *wlp2w* were caused by the same single-gene mutation (Supplementary Fig. S1). Seedlings of the *wlp2s* mutant presented a mild albino leaf phenotype at 22 °C, and a striking albino feature was observed at 28 °C. The *wlp2w* seedlings displayed an almost completely normal green leaf phenotype at 22 °C, but showed obvious albino characteristics at 28 °C. The seedlings of both mutants were absolutely white and gradually died at 32 °C (Fig. 1A–C). Both mutants showed white-striped leaves and white young panicles at the heading stage under natural high-temperature field conditions; however, the abnormal phenotype of *wlp2s* was more severe (Fig. 1D–F; Supplementary Fig. S2). Besides the differences in leaves and panicles, values of the major agronomic traits (e.g. 1000-grain weights, seed setting rates, and yields per plant) of the two mutants were significantly lower than those of wild-type plants under high-temperature field conditions (Fig. 1G–L; Supplementary Table S2). These results demonstrated that *wlp2* mutants show more severe abnormal phenotypes under high-temperature conditions, which may lead to lower biological yield.

### *wlp2* mutants present decreased chlorophyll accumulation, abnormal chloroplast development, and attenuated photosynthetic efficiency

**Fig. 2.**
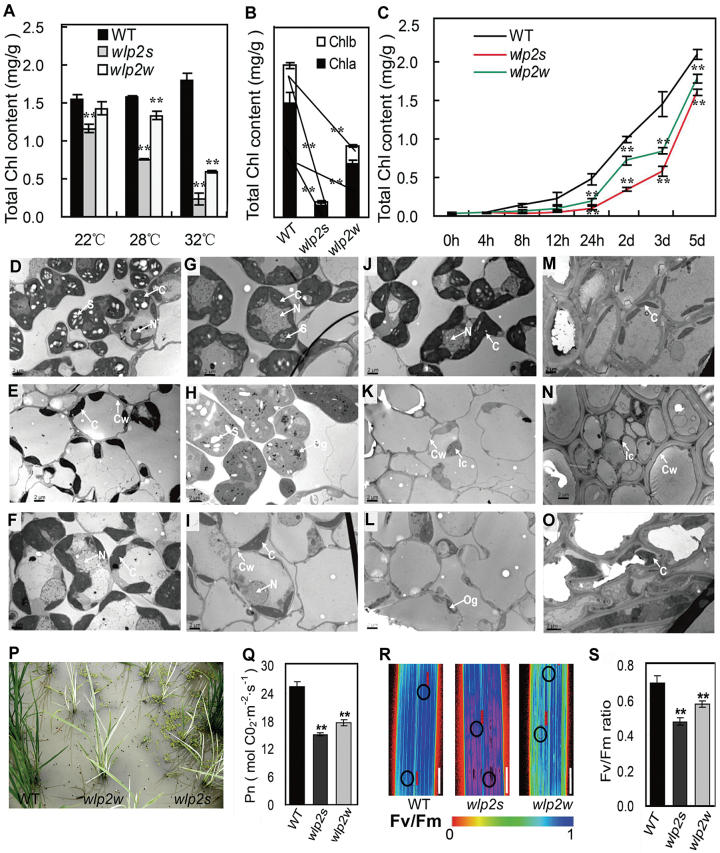
Chlorophyll content, chloroplast ultrastructure and efficiency of photosynthesis of wild-type (WT) and *wlp2* plants. (A, B) Chlorophyll content in the leaves at the four-leaf stage (A) and in young panicles at the heading stage (B). (C) Chlorophyll accumulation rates at 28 °C after cultivation in continuous dark for 7 d. (D–L) Chloroplast ultrastructures in cells of the third leaf of WT (D, G, J), *wlp2s* (E, H, K) and *wlp2w* (F, I, L) at 22 °C (D–F), 28 °C (G–I), and 32 °C (J–L). (M–O) Chloroplast ultrastructures in cells of young panicles of WT (M), *wlp2s* (N) and *wlp2w* (O) grown in field conditions (summer of 2013). C, chloroplast; N, nucleus, Og, osmiophilic plastoglobuli; Ic, immature chloroplast; S, starch granule; Cw, cell wall. (P) Phenotype of the samples used to measure photosynthetic parameters at the tillering stage. (Q) Net photosynthetic rates (Pn) of leaves of WT, *wlp2s* and *wlp2w*. (R) Color representation images of maximum photochemical efficiency (*Fv/Fm*). The color scale at the bottom of the image depicts *Fv/Fm* values; black circles represent sampling points in the leaves. Bars=1 cm. (S) *Fv/Fm* values recorded in leaves. Data in A–C, Q, and S are shown as means±SD from three individual replicates. Asterisks indicate statistical significance between WT and mutants, as determined by Student’s *t*-test: *P<0.05, **P<0.01.

Normal chloroplast biogenesis is important for plant growth, thus several closely related parameters were evaluated in wild-type plants and the two mutants. Evaluation of chlorophyll content in seedlings and young panicles showed a substantial deficiency in Chl *a*, Chl *b*, and total chlorophyll accumulation in both mutants compared with wild-type plants at 28 °C and 32 °C, however, only *wlp2s* displayed significantly lower chlorophyll accumulation at 22 °C, which was consistent with the albino phenotype observed at that temperature (Fig. 2A; Supplementary Fig. S3A, B). The chlorophyll content of young panicles in *wlp2s* and *wlp2w* was also significantly lower under natural high-temperature conditions (Fig. 2B). Moreover, analysis of the rates of chlorophyll biosynthesis at 28 °C after cultivation in continuous dark for 7 d revealed that the recovery rates of chlorophyll accumulation were slower in both mutants compared with wild-type (Fig. 2C).

Chloroplast ultrastructures were observed in third leaves of seedlings and young panicle shells. At 22 °C, we observed well-developed chloroplasts, with dense and well-structured grana lamella stacks in both wild-type and *wlp2w*, whereas fewer and smaller chloroplasts with abnormal grana lamella stacks were observed in *wlp2s* (Fig. 2D–F; Supplementary Fig. S3C–E). Under the 28 °C and 32 °C conditions, compared with wild-type plants, some mesophyll cells in both mutants presented much fewer and smaller undeveloped chloroplasts with disorganized lamellar structures and many osmiophilic plastoglobuli in young leaves (Fig. 2G–L; Supplementary Fig. S3F–K). Nonetheless, the *wlp2w* mutant also presented some chloroplasts with well-developed lamellar structures at 28 °C (Fig. 2I; Supplementary Fig. S3H). Compared with the wild-type, cells in young panicle shells of both mutants scarcely had well-developed chloroplasts, most notably in *wlp2s* under natural high-temperature conditions (Fig. 2M–O; Supplementary Fig. S3L–N).

The photosynthetic capacity of the mutants and wild-type plants grown under natural paddy conditions was also examined. This showed that net photosynthetic rate was significantly compromised in both two mutants (Fig. 2P, Q). The maximum photochemical efficiency values of PSI and PSII in both mutants were also notably decreased compared with wild-type (Fig. 2R, S). Both *wlp2* mutants emitted attenuated red chlorophyll autofluorescence compared with wild-type (Supplementary Fig. S4). These observations indicated that *wlp2* mutants suffer from defects affecting photosynthesis.

### Map-based cloning of *WLP2*

**Fig. 3.**
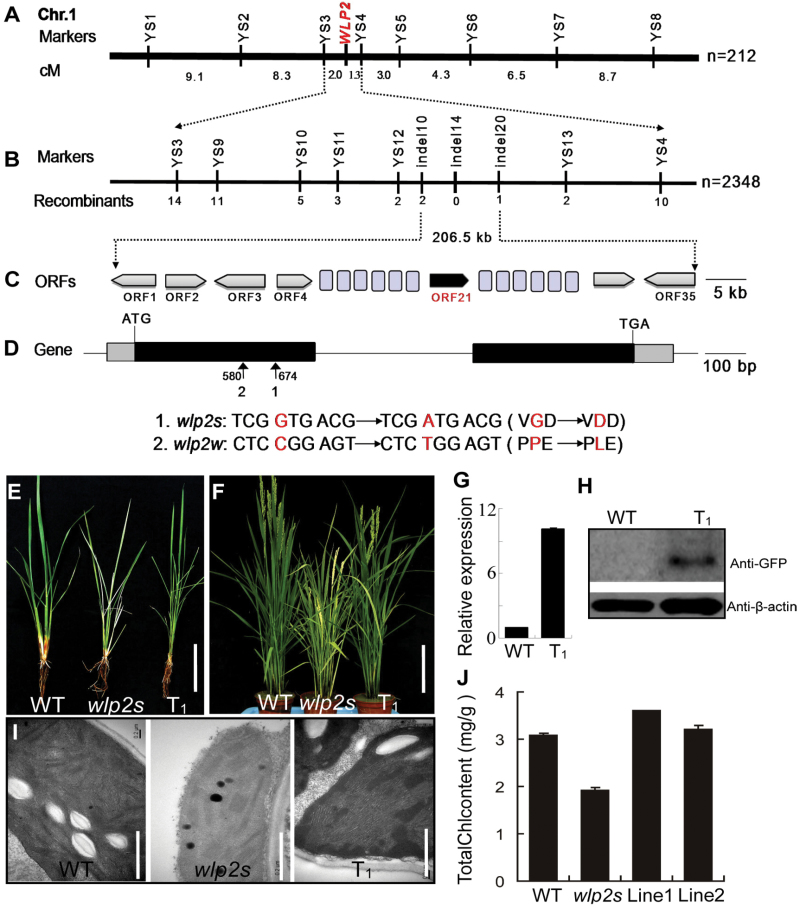
Map-based cloning of the *WLP2* gene. (A) Location of the *WLP2* locus in a 3.3 cM interval on chromosome 1. (B) The location of *WLP2* was narrowed to a 206.5 kb region. (C) Prediction of 35 putative ORFs in the 206.5 kb genomic region. (D) Gene structure of the *WLP2* (ORF21). Black boxes represent exons; the lines between them represent introns. (E, F) Phenotype of wild-type (WT), *wlp2s*, and T_1_ plants of transgenic *wlp2s* expressing *WLP2* at the seedling (E) and heading (F) stages. Bar=5 cm in E and 20 cm in F. (G, H) qRT-PCR of *WLP2* (G) and western blot analysis using GFP antibody to the WLP2-GFP fusion protein (H). (I, J) Chloroplast ultrastructures (I) and chlorophyll content of WT, *wlp2s*, and two T_1_ plants (J). Bar=5 μm in I. Data in G and J are shown as means±SD from three individual replicates.

The leaves and young panicles in F_1_ hybrids between *wlp2s* and either cv. Nanjing11 or cv. Peiai64 were similar to those of wild-type plants, and the segregation behavior in each of the two derived F_2_ populations was consistent with the Mendelian monogenic ratio of three wild-type to one albino phenotype (Supplementary Table S3). Linkage analysis fixed the location of the *WLP2* locus to a 3.3 cM interval on chromosome 1 (Fig. 3A). Based on 2348 albino plants selected from the F_2_ population derived from *wlp2s* and cv. Nanjing11, the location of *WLP2* was narrowed down to a 206.5 kb physical region (Fig. 3B). Within this region, 35 open reading frames (ORFs) were predicted (Fig. 3C). Genomic sequence analysis revealed that only the 21st ORF (LOC_Os01g63220) carried a single nucleotide transition (G→A) at position 674 bp from the ATG start codon in *wlp2s* (Fig. 3D). This entire region was also sequenced in the *wlp2w* mutant, and likewise, only ORF21 showed a nucleotide substitution (C→T) at position 580 bp from the ATG start codon in *wlp2w* (Fig. 3D). Thus, ORF21 is the candidate gene for *WLP2*.

To verify the identity of *WLP2*, the plasmid *Ubi:WLP2::GFP*, containing the wild-type *WLP2* cDNA region, was transformed into *wlp2s* and *wlp2w*. As expected, leaves and young panicles of transgenic positive plants displayed a normal phenotype in natural high-temperature conditions (Fig. 3E–H; Supplementary Fig. S5A–C). The chlorophyll content and chloroplast ultrastructure of the transgenic lines also showed normal levels (Fig. 3I, J; Supplementary Fig. S5D). Thus, ORF21 (LOC_Os01g63220) indisputably corresponds to *WLP2.*

Phylogenetic analysis revealed that *WLP2* was highly homologous with Arabidopsis *FLN1*, which encodes a putative pfkB-type carbohydrate kinase family protein fructokinase-like 1. WLP2 (OsFLN1) has a large number of homologous proteins among other higher plants, especially monocotyledons (Supplementary Fig. S6). The mutation loci of *wlp2s* and *wlp2w* occurred within the pfkB domain (Supplementary Fig. S7). Three-dimensional structures of the WLP2 protein in wild-type and the two mutants were predicted; the results indicated that the mutations in *wlp2s* and *wlp2w* introduced a structural change consisting of a variant α-helix region, suggesting that it may disturb the protein function (Supplementary Fig. S8).

### *WLP2* is mainly expressed in leaves and its protein localizes in chloroplasts

**Fig. 4.**
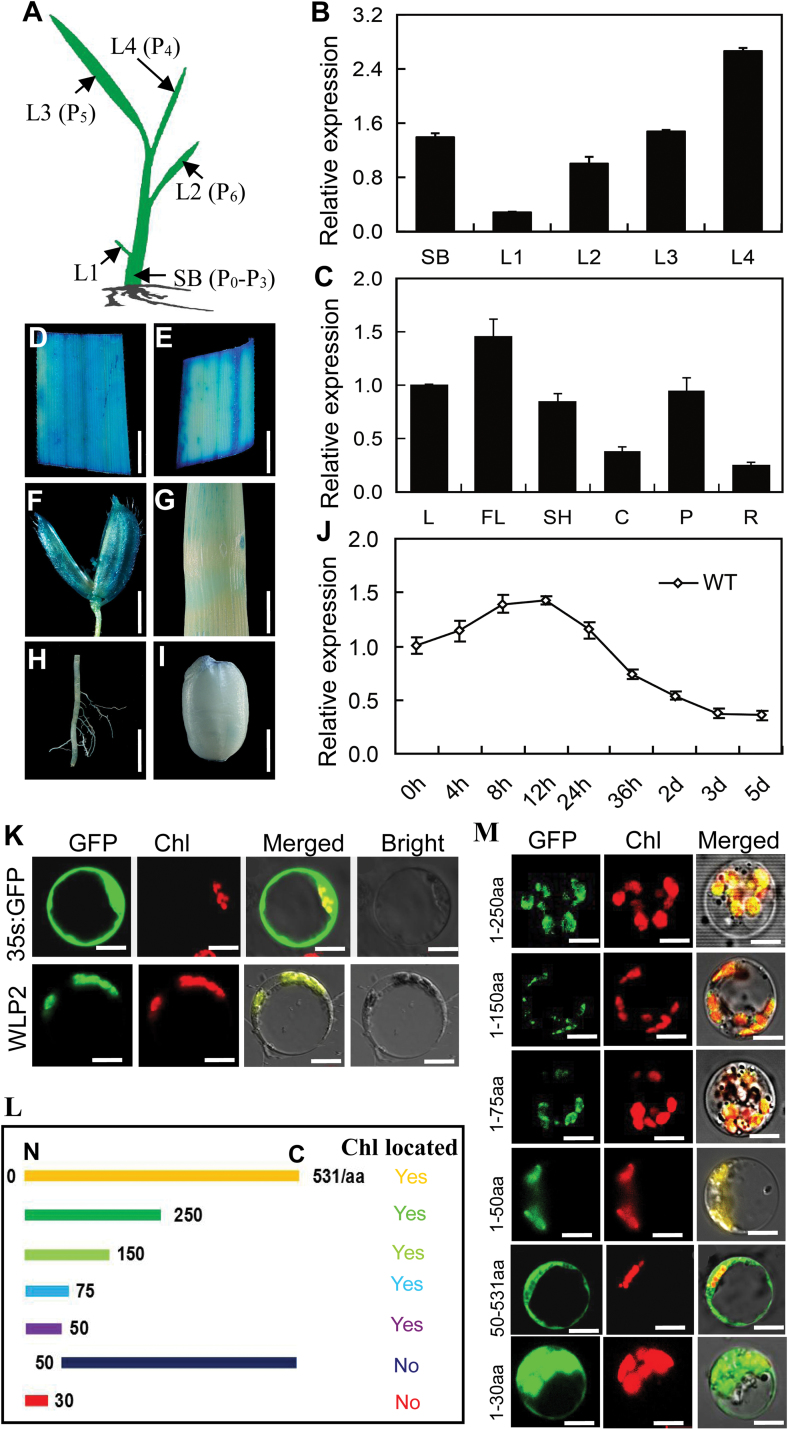
Expression pattern of *WLP2* and the subcellular localization of WLP2 protein. (A) Schematic illustration of young seedling tissues at the four-leaf stage. L1–L4 indicate the first to the fourth leaves; SB indicates the shoot base, corresponding to the P_0_–P_3_ stages of leaf development. (B) Expression pattern of *WPL2* in wild-type plants at the four-leaf stage. (C) Tissue-specific expression of *WLP2* at the heading stage in wild-type plants. RNA was isolated from basal leaves (L), flag leaves (FL), leaf sheaths (SH), culms (C), young panicles (P), and roots (R). (D–I) GUS expression in flag leaf (D), leaf sheath (E), young panicle (F), culm (G), root (H), and developing grain (I) driven by the *WLP2* promoter. Bars=5 mm in D, E, G, and H; bars=2 mm in F and I. (J) *WLP2* expression pattern in wild-type seedlings grown in continuous darkness for 7 d and subsequently exposed to light for 0, 4, 8, 12, 24, and 36 h, and 2, 3, and 5 d. Data in B, C, and J are shown as means±SD from three individual replicates. (K) GFP signal in tissues transformed with the empty GFP vector and WLP2-GFP fusion protein. Bars=5 μm. (L) Schematic diagram of the transient expression constructs containing different truncated versions of WLP2 protein. (M) GFP signals from the transient expression plasmids shown in L. The full WLP2 protein contains 531 amino acids (aa). Bars=5 μm.

Rice leaf morphological development is divided into P_0_ (leaf founder) to P_6_ (mature leaf) stages; this six-stage schema is consistent with chloroplast biogenesis (van Campen *et al.*, 2016). The expression profile of *WLP2* in various tissues of 3-week-old seedlings was investigated by qRT-PCR. The results showed that *WLP2* is expressed in all leaves, with higher levels of expression in younger leaves such as L4, which corresponds to the P_4_ stage of leaf development (Fig. 4A, B). The expression pattern of *WLP2* during the flowering stage was also investigated and the result showed that *WLP2* is constitutively expressed in the examined tissues, with higher levels in leaves, young panicles, and leaf sheaths (Fig. 4C). The expression profile was also investigated by transforming rice with the *GUS* reporter gene driven by the *WLP2* promoter. Histochemical analysis of transgenic plants showed that GUS activity accumulated more in leaves, young panicles, and leaf sheaths, consistent with the qRT-PCR findings (Fig. 4D–I). Wild-type seedlings were grown under continuous darkness and subsequently exposed to light. The expression of *WLP2* was strongly induced after 4 h of illumination and peaked after 12 h of illumination. It then gradually decreased over time and returned to the low basal level (Fig. 4J). These observations indicate that *WLP2* likely plays a role in the light regulation of chloroplast development.

To identify the subcellular localization of WLP2 protein, a transient expression system was constructed by delivering the *35S:WLP2::GFP* construct into rice protoplasts. The results showed that the GFP signal was localized in chloroplasts (Fig. 4K). To estimate the WLP2 chloroplast-targeting signal region, six further transient expression genetic constructs containing different amino acid fragments of WLP2 were also transformed into rice protoplasts (Fig. 4L). The GFP signals of all fusion plasmids with different lengths of WLP2, even WLP2^1–50aa^, showed the typical chloroplast localization pattern, except for WLP2^1–30aa^ and WLP2^50–531aa^, which displayed a similar signal pattern to that of the *35S:GFP* control plastid (Fig. 4M). These results imply that the N-terminal 50 amino acids are both necessary and sufficient for WLP2 to be targeted to the chloroplast.

### Expression levels of chloroplast-associated genes are affected in the two mutants

**Fig. 5.**
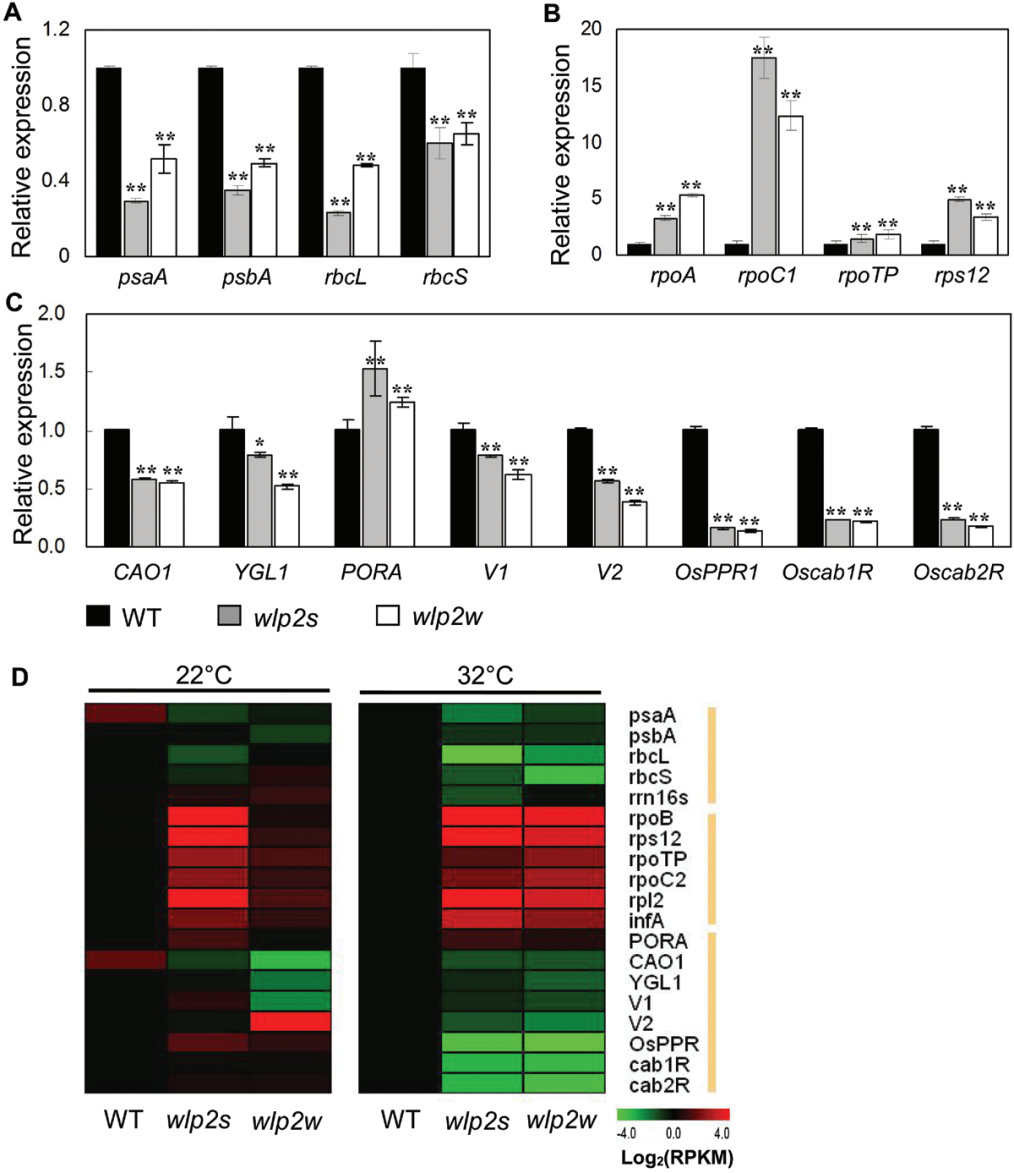
Transcriptional changes of chloroplast-associated genes in *wlp2* mutants. (A–C) Expression levels of PEP-dependent genes (A), NEP-dependent genes (B) and photosynthesis-associated genes (C) in 3-week-old seedlings of wild-type (WT), *wlp2s*, and *wlp2w* grown at 28 °C. (D) Heat map of gene expression in plants grown at 22 °C and 32 °C; the gene expression of WT plants represents a relative standard (Log_2_(RPKM)=0) at 32 °C. Data are shown as means±SD from three individual replicates.

Transcript levels of chloroplast-associated genes were investigated in seedlings of the two mutants grown at 28 °C. Expression levels of PEP-dependent photosynthesis genes, such as *psaA*, *psbA*, *rbcL*, and *rbcS,* were significantly decreased in both *wlp2s* and *wlp2w* compared with the wild-type (Fig. 5A). In contrast, the NEP-dependent genes, including the subunit RNA polymerase genes (*rpoA*, *rpoC1*, and *rpoTp*), were significantly increased in both two mutants (Fig. 5B). Expression levels of other genes associated with chlorophyll biosynthesis (*CAO1*, *YGL1*), chloroplast development (*V1*, *V2*), and photosynthesis (*Oscab1R*, *Oscab2R*) were also down-regulated in the two mutants (Fig. 5C).

The expression levels of the abovementioned genes were also investigated in plants grown under 22 °C and 32 °C conditions. A heat map illustrating gene transcription levels (Fig. 5D) shows that the trends of differences in gene expression levels between wild-type and the two mutants were similar to those observed under 28 °C conditions, and that the genes were up- or down-regulated more strongly at 32 °C than at 22 °C. These findings suggest that abnormal expression of these chloroplast-associated genes may be responsible for the albino phenotype of *wlp2s* and *wlp2w*, especially under high-temperature conditions.

### WLP2 and its paralog OsFLN2 interact with OsTRXz

**Fig. 6.**
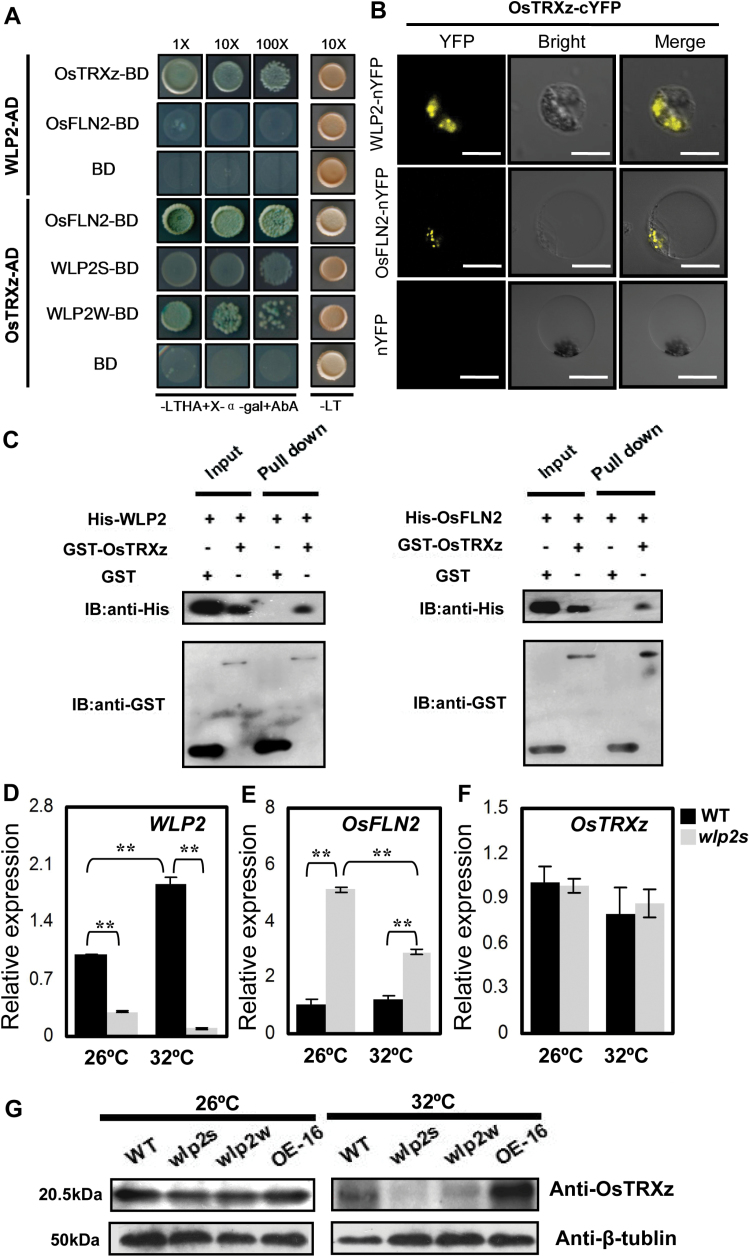
WLP2, OsFLN2, and OsTRXz form a TRX-FLN regulatory module in the PEP complex. (A) Interaction of WLP2, OsTRXz, and OsFLN2 in a yeast two-hybrid assay. Yeast transformants were spotted on to control medium (SD/-Leu/-Trp [-LT]) and selective medium (SD/-Leu/-Trp/-His/-Ade [-LTHA] plus X-α-gal and Aureobasidin A [AbA]). (B) A bimolecular fluorescence complementation assay showed that OsTRXz can interact with WLP2 and OsFLN2 in rice protoplast cells. Bars=5 μm. (C) *In vitro* pull-down assay of WLP2, OsFLN2, and OsTRXz. The fusion proteins of WLP2 and OsFLN2 with a His tag (His-WLP2, His-OsFLN2) and OsTRXz with a GST tag (GST-OsTRXz) were detected by anti-His antibody and anti-GST antibody, respectively. (D–F) Levels of mRNA of *WLP2* (D), *OsFLN2* (E), and *OsTRXz* (F) under normal (26 °C) or high (32 °C) temperature conditions. (G) Immunoblot analyses of OsTRXz in wild-type (WT), *wlp2* mutants, and *WLP2* overexpression line 16 (OE-16) in seedlings at the third-leaf stage maintained at 26 °C and 32 °C.

The interactions between WLP2 (OsFLN1), OsFLN2, and OsTRXz were examined by yeast two-hybrid and bimolecular fluorescence complementation assays *in vivo* and a pull-down assay *in vitro*. The results showed that WLP2 and its paralog OsFLN2 can interact with OsTRXz (Fig. 6A–C). However, WLP2 and OsFLN2 cannot interact with each other (Fig. 6A). Furthermore, OsTRXz can interact with both WLP2S and WLP2W (Fig. 6A). These results suggest that there is also a TRX-FLN interaction module for the regulation of PEP activity in rice similar to that described in Arabidopsis.

To further explore the relationship between OsFLN1 and OsFLN2 in the function of the TRX-FLN regulatory complex, expression levels of *WLP2*, *OsFLN2*, and *OsTRXz* were examined in *wlp2s* and wild-type plants grown under different temperature conditions. Under both normal (26 °C) and high-temperature (32 °C) conditions, the mRNA level of *WLP2* was lower and the level of *OsFLN2* was higher in *wlp2s* compared with wild-type (Fig. 6D, E), indicating that these genes may have complementary functions in the TRX-FLN complex. Although the levels of *OsTRXz* mRNA did not dramatically change in either condition (Fig. 6F), the level of OsTRXz protein was obviously attenuated in *wlp2* mutants under heat stress (32 °C) (Fig. 6G). However, no significant differences in protein levels were found among the wild-type plants, the two mutants, and the overexpression (OE) line (OE-16) at normal temperature (Fig. 6G). These results further validate that WLP2 maintains the stability of OsTRXz and the function of the TRX-FLN complex under heat stress.

### *wlp2* mutants show heat-stress-induced death with elevated ROS accumulation

**Fig. 7.**
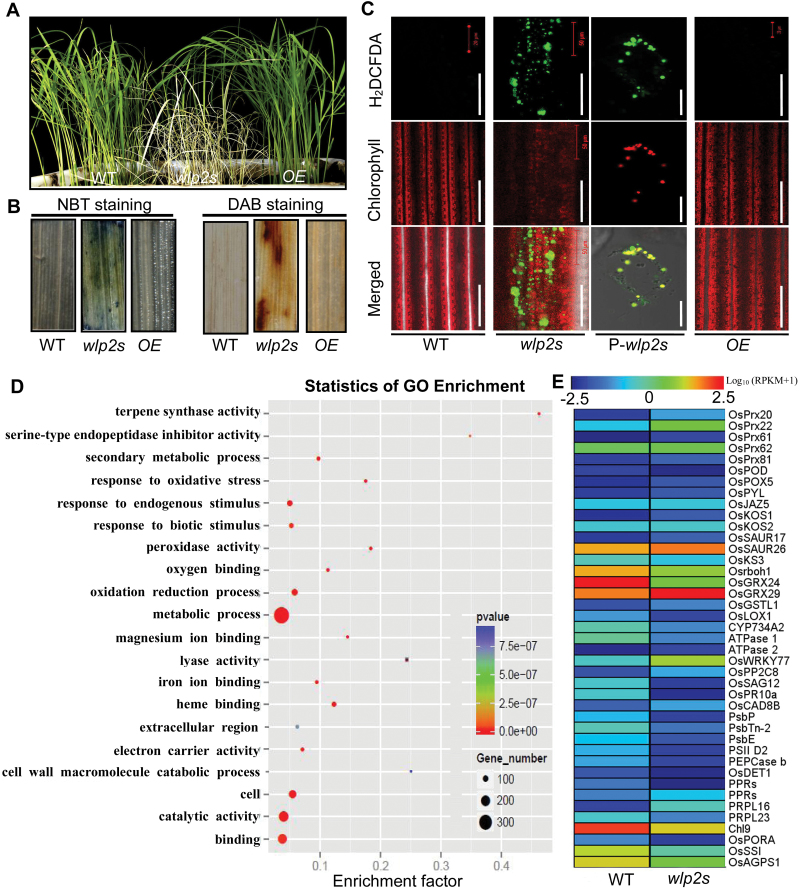
The *wlp2* mutants showed heat-stress-induced death with elevated ROS accumulation. (A) Phenotype of wild-type (WT) and *wlp2s* plants at the four-leaf stage grown under heat stress (32 °C). (B) NBT and DAB staining in blades of 2-week-old seedlings in WT, *wlp2s*, and a *WLP2* overexpression line (OE) grown at 32 °C. (C) Microscopic analysis of leaves of 2-week-old seedlings of WT, *wlp2s*, and *WLP2* OE lines, and protoplast cells of *wlp2s* (P-*wlp2s*), incubated with H_2_DCFDA at 32 °C. Green staining represents oxidized H_2_DCFDA and red represents chlorophyll. H_2_DCFDA foci co-localize with chloroplasts in P-*wlp2s*. Bars=50 μm in WT, *wlp2s*, OE; bar=2 μm in P-*wlp2s*. (D, E) RNA-seq analysis of *wlp2s* and WT grown under the heat stress (32 °C) condition. (D) GO enrichment analysis of differentially expressed genes (DEGs) between *wlp2s* and WT (*P*≤0.05). (E) Expression heat map of selected DEGs.

The *wlp2* mutants showed spontaneous heat-stress-dependent death when grown under the 32 °C condition (Fig. 7A). Production of O_2_^–^ and H_2_O_2_ in 3-week-old leaves was detected using NBT and DAB staining, respectively. More densely shadowed coloration over the surface of *wlp2s* leaves suggested that they accumulated more O_2_^–^ and H_2_O_2_ than the wild-type and OE lines (Fig. 7B). The leaves of two mutants, the wild-type and an OE line were also incubated in the presence of the general oxidative stress fluorescent probe H_2_DCFDA to assess the redox balance and ROS accumulation. Microscopic detection of the oxidized fluorescent form of the probe molecule revealed ROS-generating foci in both mutants, but not in the wild-type or the OE line. Moreover, the fluorescent signal of ROS was mainly located in the chloroplasts (Fig. 7C; Supplementary Fig. S9A), suggesting that the redox balance in chloroplasts of *wlp2* mutants was broken under the heat-stress condition. Furthermore, we also found excess ROS production in *wlp2s* suffering heat stress under natural field conditions (Supplementary Fig. S9B).

To test whether the elevated ROS levels can give rise to nuclear genetic reprogramming, enabling the plant to adapt to heat stress, RNA-seq analysis of *wlp2s* and wild-type plants grown under the 32 °C condition was performed. Compared with wild-type, a total of 874 differentially expressed genes were identified in *wlp2s*, including 530 transcripts that were up-regulated and 344 transcripts that were down-regulated relative to wild-type. Based on GO analysis, these genes were classified into different biological processes or molecular functions (Fig. 7D; Supplementary Fig. S9C). Interestingly, many genes associated with the GO terms metabolic process, oxidative stress, oxidation-reduction process, and abiotic stimulus were found to be up- or down-regulated in *wlp2s* (Fig. 7D, E). For example, the expression levels of the stress-associated genes *OsPrx20*, *OsPrx22*, *OsPrx62*, *OsPOD, OsPOX5*, and *OsLOX1*, NADPH oxidase and glutaredoxin genes *Osrboh1*, *OsGRX24*, and *OsGRX29*, and many photosynthesis, chloroplast development, chlorophyll synthesis, or plastid starch metabolism genes, such as *PsbP*, *PsbE*, *PSII-D2*, *PRPs*, *Chl9*, *OsSSI*, and *OsAGPS1*, were all dramatically changed in *wlp2s* compared with wild-type (Fig. 7E). Furthermore, mRNA levels of genes encoding ascorbate peroxidase and glutaredoxin (two major scavenger enzymes in the ROS degradation pathway) were mostly up-regulated as indicated by qRT-PCR (Supplementary Fig. S9D).

## Discussion

### *WLP2* is involved in the regulation of PEP-mediated transcription during chloroplast biogenesis

Many leaf-color mutants show abnormal phenotypes in response to temperature changes (Kusumi *et al.*, 2011; Wang *et al.*, 2016*c*). Here, the two allelic mutants *wlp2s* and *wlp2w* exhibited albino leaves and did not survive at high temperatures (Fig. 1). Furthermore, chlorophyll accumulation, chloroplast structure, photosynthetic efficiency, and major agronomic characteristics of both mutants were greatly impaired under natural high-temperature conditions (Fig. 2; Supplementary Table S2). However, the mutants showed no obvious leaf color changes under cold or excess-light stress conditions (Supplementary Fig. S10). These results indicate that *wlp2s* and *wlp2w* are two typical high-temperature-sensitive rice mutants with abnormal chloroplast biogenesis and disordered agronomic characteristics.

**Fig. 8.**
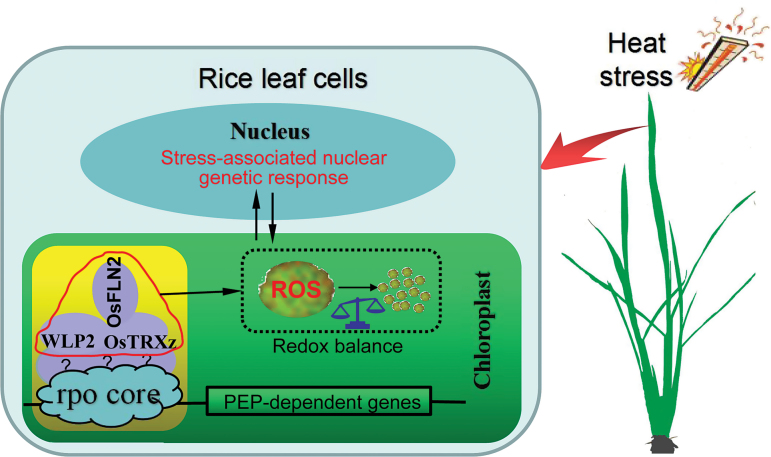
Schematic illustration of the function of TRX-FLN in the regulation of redox balance and transcription of PEP-dependent genes. TRX-FLN regulates the expression of PEP-encoded genes, further impacting on chloroplast development; it may also act upon redox balance through affecting ROS activities under heat stress. Furthermore, elevated ROS can give rise to nuclear genetic reprogramming, enabling the plant to adapt to particular heat stress. The yellow box represents the PEP complex; the red circled complex represents the TRX-FLN regulatory module; the black dotted box indicates the redox balance in chloroplasts; and the rpo core represents subunits of the PEP complex.

The PEP-mediated chloroplast transcriptional machinery is indispensable to the maintenance of highly efficient photosynthesis, which is essential for the plant’s growth (Demarsy *et al.*, 2006; Kusumi and Iba, 2014). In Arabidopsis, many PAP mutants associated with this transcriptional machinery have been identified, such as *pap1*, *pap2*, *pap3*, *pap5*, *pap6*, and *pap10*, in which the transcription levels of NEP-mediated genes were elevated and transcription levels of PEP-mediated genes were decreased (Arsova *et al.*, 2010; Gilkerson *et al.*, 2012; Pfalz *et al.*, 2006). Here, we identified a novel PEP-associated protein, WLP2, which is a homolog of AtFLN1 (PAP6) in Arabidopsis (Fig. 3; Supplementary Fig. S7). *WLP2* is highly expressed at the P_4_ stage, which corresponds to the activation of the photosynthetic apparatus (Fig. 4A, B). Furthermore, the two mutants of *WLP2* presented elevated expression of NEP-mediated genes and reduced expression of the photosynthesis-associated genes transcribed by PEP (Fig. 5). Accordingly, as a novel rice PAP of the PEP complex, *WLP2* plays an essential role in chloroplast development and photosynthesis in rice (Fig. 8).

### A TRX-FLN regulatory module mediates PEP activity and chloroplast redox balance under heat stress

PEP is the dominating RNA polymerase in mature chloroplasts, and the mechanism of regulation of its activity is complicated and elusive (Kremnev and Strand, 2014; Pfalz *et al.*, 2006). In Arabidopsis, thioredoxin Z (TRXz) and its two target proteins FLN1 and FLN2 can form a complex that regulates PEP activity during PEP-dependent transcription. The *trxz* mutant displays a severe albino phenotype. The *fln1* mutant results in an albino phenotype, *fln2* plants display chlorosis but can revert to green color as they continue to grow, and *fln1 fln2* double mutants have a similar but more severe phenotype than either single mutant (Arsova *et al.*, 2010; Gilkerson *et al.*, 2012; Wimmelbacher and Börnke, 2014), indicating that FLN1 may play a more important role than FLN2 and that it is indispensable in the FLN functions of the regulatory complex. Our findings show that although WLP2 cannot interact with OsFLN2 (FLN) in rice, both WLP2 and OsFLN2 can interact with OsTRXz (TRX) (Fig. 6), suggesting that they can also form a TRX-FLN regulatory module for the regulation of PEP activity, similar to that described in Arabidopsis (Fig. 8). Furthermore, the level of *OsFLN2* mRNA increases, with a decrease of *OsFLN1* expression, in *wlp2s* at normal temperatures, whereas this compensation is significantly attenuated under heat stress (Fig. 6D, E). These findings suggest that the transcription levels of *OsFLN1* and *OsFLN2* maintain a relative dynamic balance in regulating PEP activity; OsFLN1 plays the major FLN function in the TRX-FLN complex, especially under heat stress, and OsFLN2 may partially replace the function of OsFLN1 at normal but not high temperatures, which explains the more abnormal phenotypes observed in *wlp2* mutants under heat stress.

Plastid thioredoxins are involved in protecting plastids against oxidative damage by regulating the redox balance (Bohrer *et al.*, 2012; Collin *et al.*, 2003; Montrichard *et al.*, 2009). So far, few plastid thioredoxin family proteins have been identified in rice. OsTRXm is involved in the regulation of activity of a target peroxiredoxin (Prx) through the reduction of Cys disulfide bridges. *OsTrxm* RNAi plants showed pale green leaves with increasing ROS production (Chi *et al.*, 2008). OsTRXz interacts with OsCHL1, which encodes a Mg-chelatase I subunit, to become involved in chlorophyll synthesis (Zhang *et al.*, 2015). Recently, Sun *et al.* (2017) found that OsTRXz can interact with TSV, a putative plastidic oxidoreductase, to protect chloroplast development under cold stress, and that knockdown of OsTRXz also caused albino death in rice. Here, we found that OsTRXz can interact with WLP2 and its paralog OsFLN2 to form a TRX-FLN module (Fig 6A–C). The mutants of *WLP2* show heat-stress-induced albino lethality and elevated ROS production in chloroplasts (Fig. 7A), implying an impaired redox balance in chloroplasts. Furthermore, WLP2 can maintain OsTRXz stability and TRX-FLN function to protect the chloroplast from heat stress (Fig. 6D–G), which is similar to the function of TSV under cold stress (Sun *et al.*, 2017). However, extensive study is still necessary in order to exploit the process by which the TRX-FLN module guides the redox balance in chloroplasts, especially under heat stress.

### Potential advantages of utilizing *wlp2w* as an early selective marker for enhancing seed purity and mechanical production of hybrid rice

Male sterility (MS) lines are an important germplasm resource for hybrid breeding systems. In this study, we found that *wlp2w* showed albino lethality under high-temperature conditions, but no discernible differences in the main agronomic traits were observed at normal temperature (Supplementary Fig. S1; Supplementary Table S1). Thus, we anticipated that the albino phenotype of the *wlp2w* mutant could be used as an early marker for enhancing seed purity and the mechanized production of hybrid rice. Through continuous backcrossing, self-pollination, and molecular-marker-assisted selection, the *wlp2w* mutant gene has been imported into the MS line Yu01s (BC_2_F_2_). During the production of the Yu01s^*wlp2w*^ line by self-pollination, or production of hybrid seeds by cross-pollination, we could easily eliminate false MS seedlings to enhance MS or hybrid seed purity (Supplementary Fig. S11A). Furthermore, the purity of commercial F_1_ hybrid seeds derived from the cross MS Yu01s^*wlp2w*^ and the restorer line can be easily assessed before they are sold to farmers (Supplementary Fig. S11B). In addition, achieving mechanical production of hybrid rice seeds is challenging. In an experiment addressing this issue, we attempted to transfer the *wlp2w* mutant gene into restorer lines and to sow the mixed parent seeds (the normal green sterile and restorer lines) in proper proportions, transplant, cross-pollinate, and harvest them using a machine. When the hybrid seeds were germinated and grown in a rice nursery factory at a constant temperature of 32 °C, all the non-hybrid seeds from self-pollination of restorer lines were albino lethal, whereas the true hybrid seeds grew normally (Supplementary Fig. S11C, D). Using this strategy, a whole-course automated production of hybrid seeds could be developed in the future.

## Supplementary data

Supplementary data are available at *JXB* online.

Fig. S1. Allelism test for the abnormal phenotypes of *wlp2s* and *wlp2w* by hybridization.

Fig. S2. Phenotypes of wild-type and *wlp2* plants at the heading stage during different years.

Fig. S3. Chlorophyll content and chloroplast ultrastructure of wild-type and *wlp2* mutant plants.

Fig. S4. Chlorophyll autofluorescence analysis of wild-type and *wlp2* mutant plants.

Fig. S5. Phenotypes of wild-type, *wlp2w*, and transgenic positive T_1_ plants.

Fig. S6. Phylogenetic tree of WLP2 proteins.

Fig. S7. Alignment of amino acid sequences of WLP2 homologous proteins from higher plants.

Fig. S8. Predicted 3D protein structures of WLP2 protein between wild-type and the two *wlp2* mutants.

Fig. S9. ROS production induced by heat stress and RNA-seq analysis in *wlp2* mutants.

Fig. S10. The responses of *wlp2s* and wild-type plants to other abiotic stresses.

Fig. S11. Potential use of *wlp2w* as an early selective marker for enhancing seed purity and automated production of hybrid rice.

Table S1. Primer sequences used in this study.

Table S2. Main agronomic traits of wild-type, *wlp2s*, and *wlp2w* plants grown at the Hangzhou paddy field in 2014 and 2015.

Table S3. Genetic analysis of the *wlp2s* mutant gene.

## Supplementary Material

supplementary_Figs_S1_S11_Tables_S1_S3
