# Supplementary material for: White Leaf and Panicle 2, encoding a PEP-associated protein, is required for chloroplast biogenesis under heat stress in rice
Source: J Exp Bot. 2017 Oct 16;68(18):5147–60. doi: 10.1093/jxb/erx332 (PMC5853965; doi:10.1093/jxb/erx332)
Supplement: supplementary_Figs_S1_S11_Tables_S1_S3 [file erx332_suppl_supplementary_figs_s1_s11_tables_s1_s3.pdf]

White Leaf and Panicle 2 **encoding a PEP-associated protein, is required for chloroplast biogenesis under heat stress in rice (*Oryza sativa* L.)**

Yusong Lv[1, 2, †], Gaoneng Shao[1, †], Jiehua Qiu[1], Guiai Jiao[1], Zhonghua Sheng[1], Lihong Xie[1], Yawen Wu[1], Shaoqing Tang[1], Xiangjin Wei [1, *], Peisong Hu[1, *]

[1]State Key Laboratory of Rice Biology, China National Rice Research Institute, Hangzhou, 310006, China

[2]National Key Laboratory of Crop Genetic Improvement, Huazhong Agricultural University, Wuhan, 430070, China

[*] Corresponding. peisonghu@126.com, hupeisong@caas.cn (P. Hu); weixiangjin@caas.cn (X. Wei)

[†]These authors contributed equally to this work.

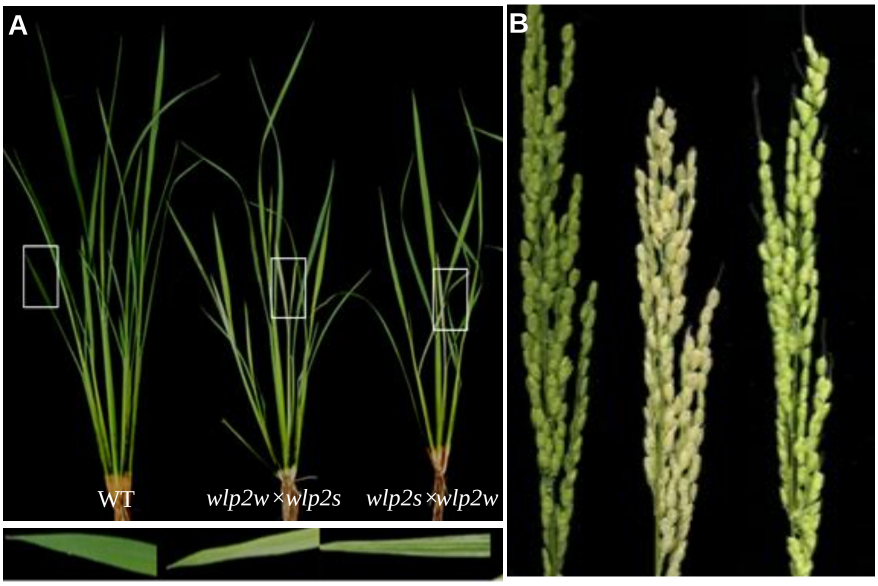

Fig. S1. Allelism test for the abnormal phenotypes of the *wlp2s* and *wlp2w* by hybridization. (A) Phenotypes of F_1 hybrid plants crossed by *wlp2s* and *wlp2w* at the tillering stage; white boxes represent basal leaves of the wild-type and F_1 hybrid plants. (B) Young panicles of F_1 hybrid plants. The wild-type and F_1 hybrid plants were grown in paddy fields during the normal growing seasons at the China National Rice Research Institute, in Hangzhou.

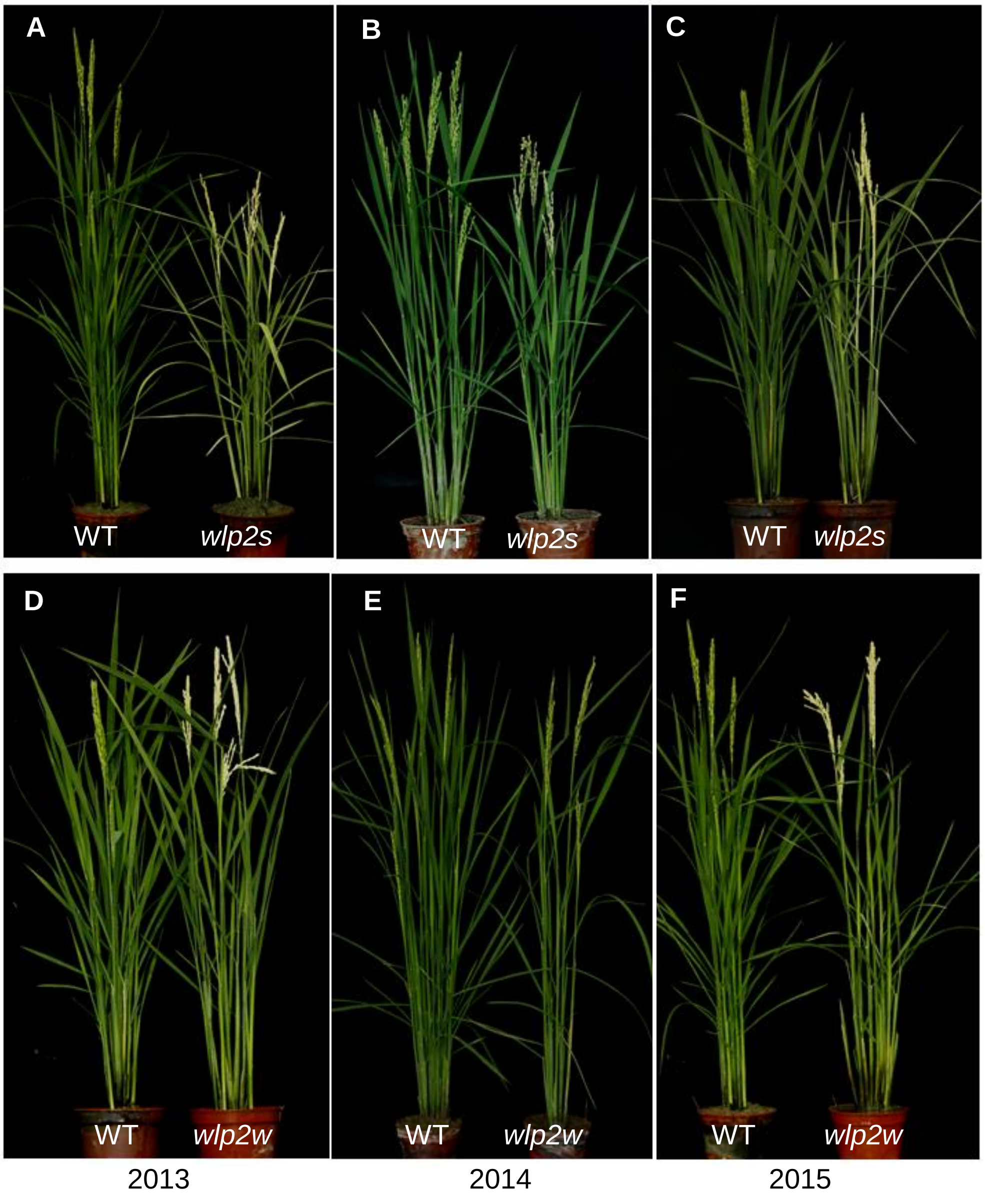

Fig. S2. Phenotypes of wild-type and *wlp2* plants at the heading stage during different years. The wild-type and *wlp2* plants were grown in paddy fields during the normal growing seasons in 2013 (A, D), 2014 (B, E) and 2015 (C, F) at the China National Rice Research Institute, in Hangzhou (30 °N latitude). The mean temperature at the heading stage in the summer of 2013, 2014 and 2015 were 34.5 ℃, 28.5 ℃ and 32.8 ℃, respectively.

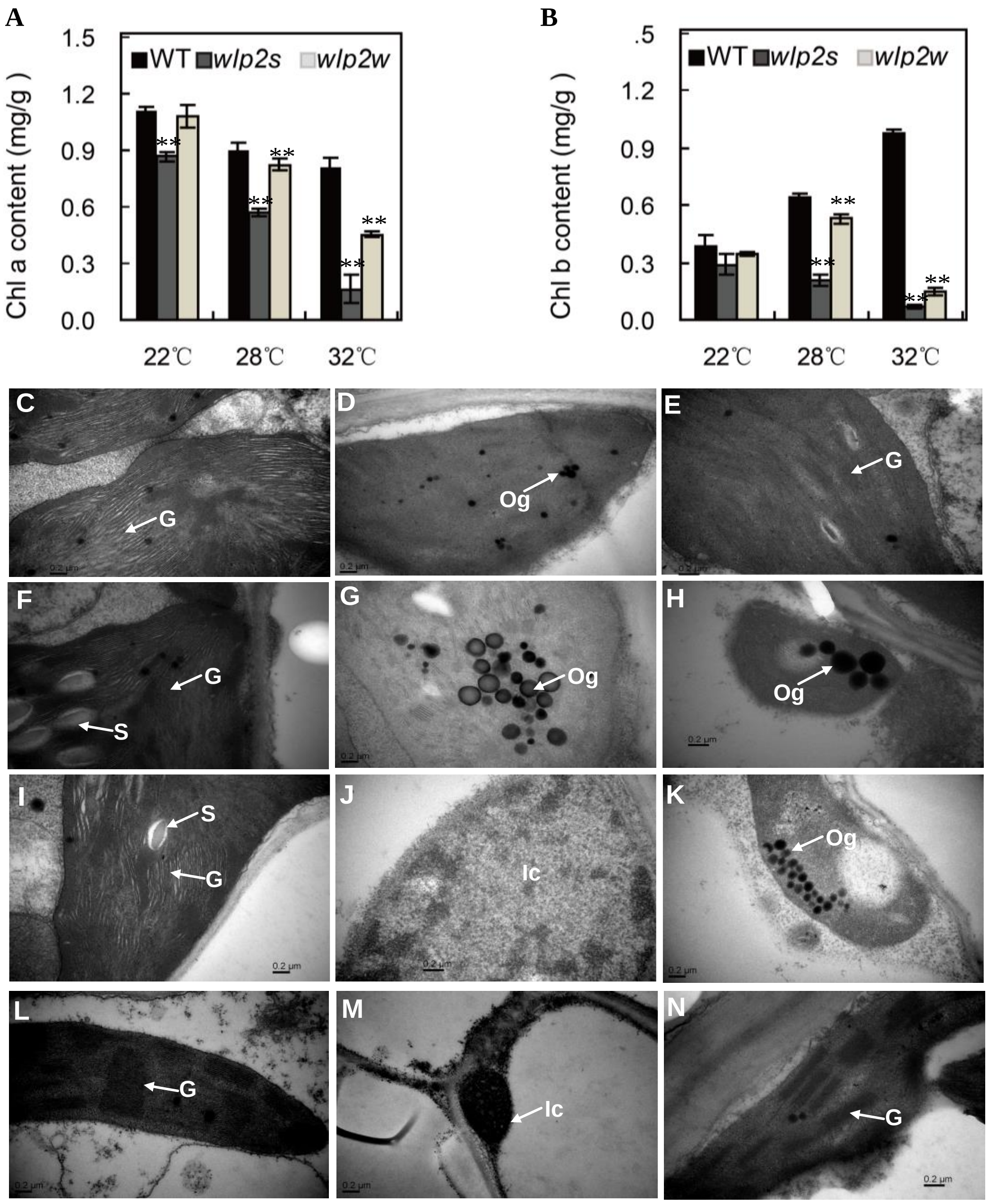

Fig. S3. Chlorophyll content and chloroplast ultra-structure of wild-type and *wlp2* mutant plants. (A, B) Chl a, b in leaves of wild-type, *wlp2s* and *wlp2w* at the four-leaf stage. (C-K) The ultrastructures of chloroplasts in lls of the third leaf of wild-type (C, F, I), *wlp2s* (D, G, J) and *wlp2w* (E, H, K) at 22 ℃ (C-E), 28 ℃ (F-H), 32 ℃ (I-K). (L-N) The ultrastructures of chloroplasts in ells of young panicle of wild-type (L), *wlp2s* (M) and *wlp2w* (N) under field condition in Hangzhou (2013). C, chloroplast; G, granna stacks; N, nucleus, Og, osmiophilic plastoglobuli. Ic, immature chloroplast; S, starch granule. Data in (A, B) are shown as means ± SD from three individual replicates. The asterisks indicate statistical significance between the wild-type and the mutants, as determined by the Student's *t*–test (** $P < 0.01$).

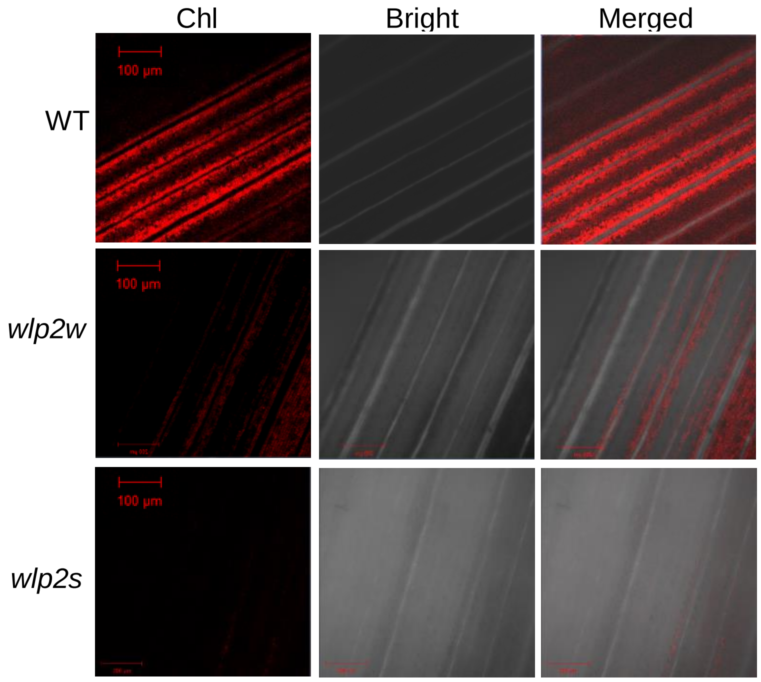

Fig. S4. Chlorophyll autofluorescence analysis of the wild type and *wlp2* mutants. Chlorophyll autofluorescence was measured as an indicator of PSII integrity in leaves of wild-type, *wlp2s* and *wlp2w* plants.

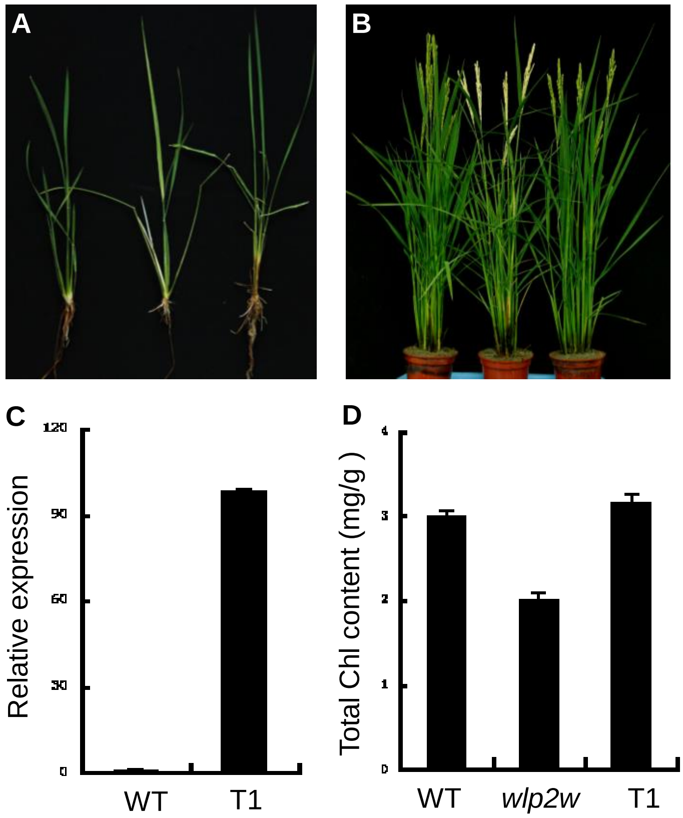

Fig. S5. Phenotypes of wild-type, *wlp2w* and transgenic positive T_1 plants. (A, B) Phenotype of wild-type, *wlp2w* and transgenic positive T_1 plants (from left to right) at the tillering stage (A) and heading (B) stages. (C) Expression levels of *WLP2* in wild-type and transgenic positive T_1 plants. (D) Chlorophyll content in 3-weeks seedlings. Data in (C, D) are shown as means ± SD from three individual replicates.

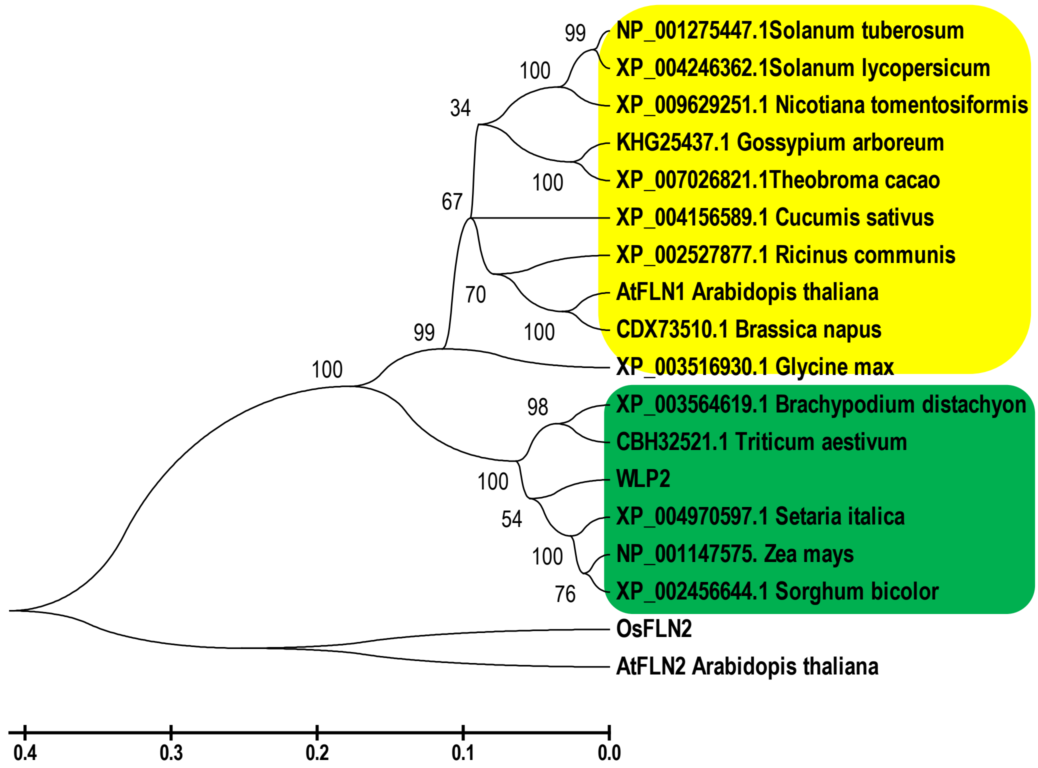

Fig. S6. Phylogenetic tree of WLP2 proteins. The phylogenetic tree was constructed using by MEGA 4.0 program and the neighbor joining algorithm. Bootstrap values are shown at each node. Bar indicator of genetic distance is based on branch length. The yellow box stands for dicotyledons and the green box represents monocotyledons.

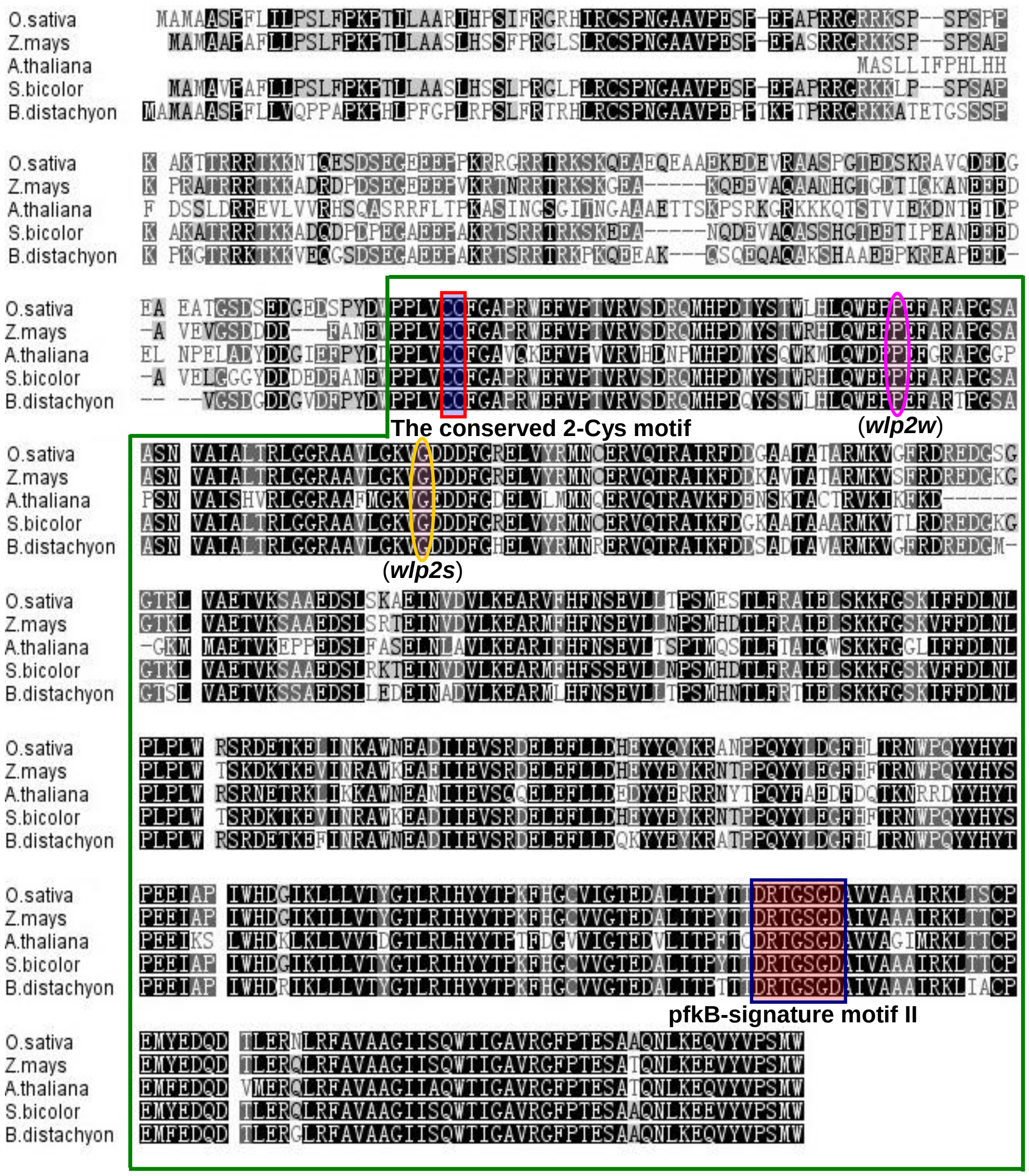

Fig. S7. Alignment of amino acid sequences of WLP2 homologous proteins from higher plants. Black shading indicates identical residues and gray shading similar residues. The comparison was aligned by CLUSTAL W. The green box represents the pfkB domain, the red box indicates the conserved double cysteine motif, and the blue box the pfkB-signature motif II. The oval boxes indicate the mutation sites of *wlp2s* and *wlp2w*. RefSeq numbers: O.sativa, XP_015616764.1; Z.mays, NP_001147575; A.thaliana, NP_190977.1; S.bicolor, XP_002456644.1; B.distachyon, XP_003564619.1.

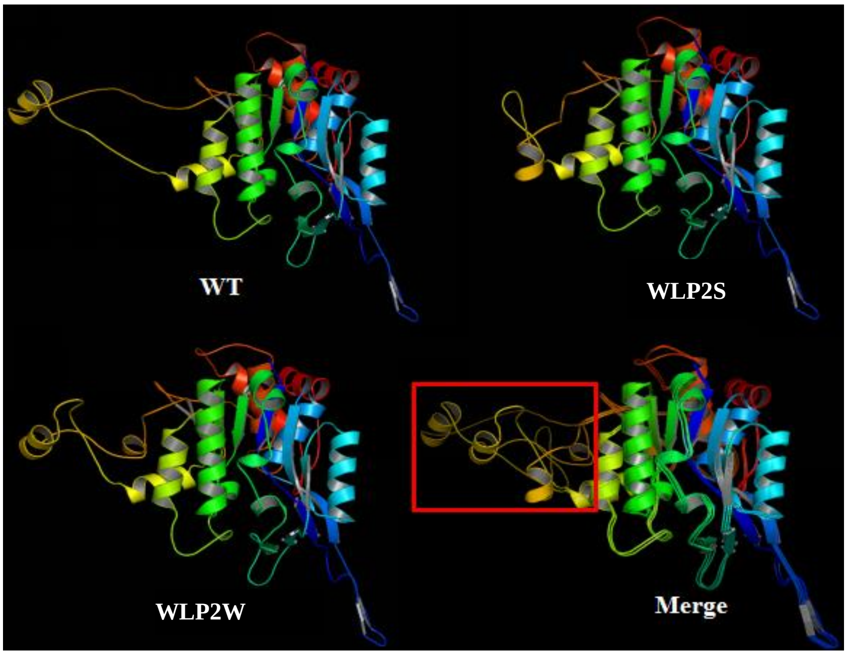

Fig. S8. Predicted 3D protein structures of WLP2 protein in the wild-type and the two *wlp2* mutants. Protein 3D structures were predicted by SWISS-MODEL (http://swissmodel.expasy.org/). The red box represents the region with 3D structure differences between wild type and the mutants

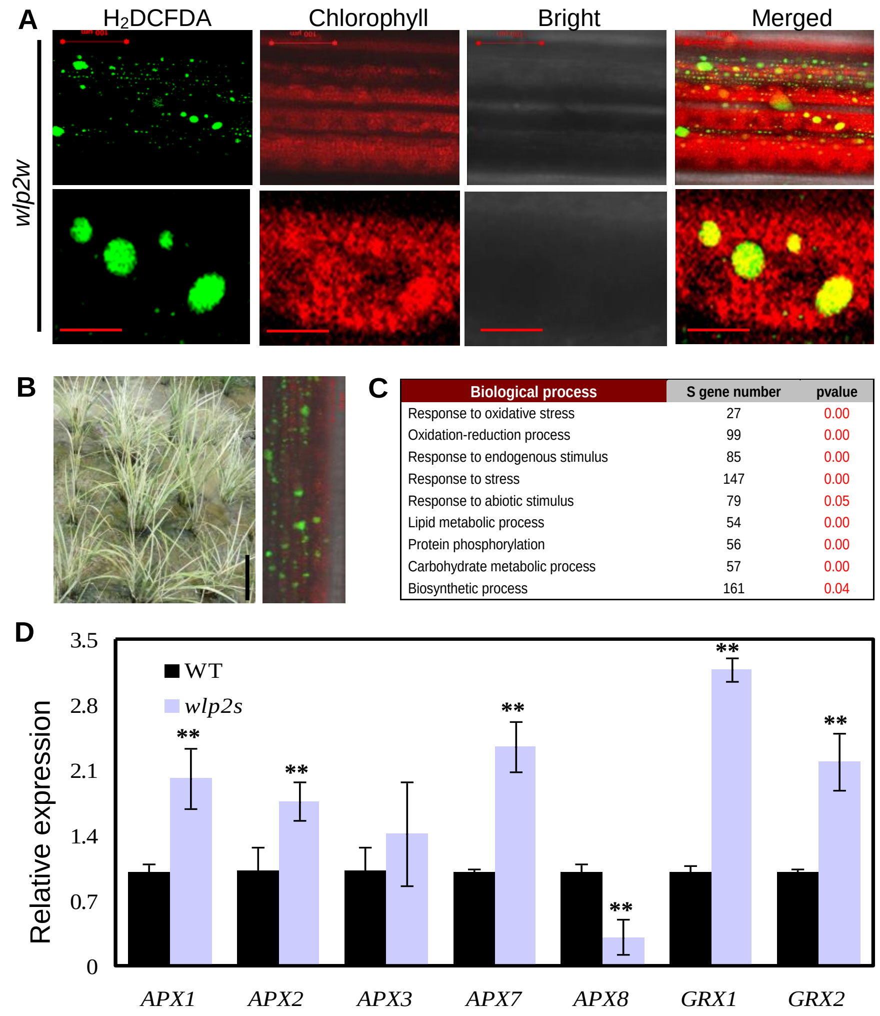

Fig. S9. ROS production induced by heat stress and RNA-seq analysis in *wlp2* mutants. (A) Microscopic analysis of leaves of two-week-old seedlings of *wlp2w* incubated with H_2DCFDA. Oxidized H_2DCFDA is represented in green staining and chlorophyll in red. (B) The excess produced ROS indicated redox inbalance in *wlp2s* when the mutant was suffered from heat stress under natural field conditions. (C) List of GO terms indicating the large number of genes associated with responses to abiotic stress. (D) The expression levels of genes associated with ascorbate peroxidase and glutaredoxin synthesis.

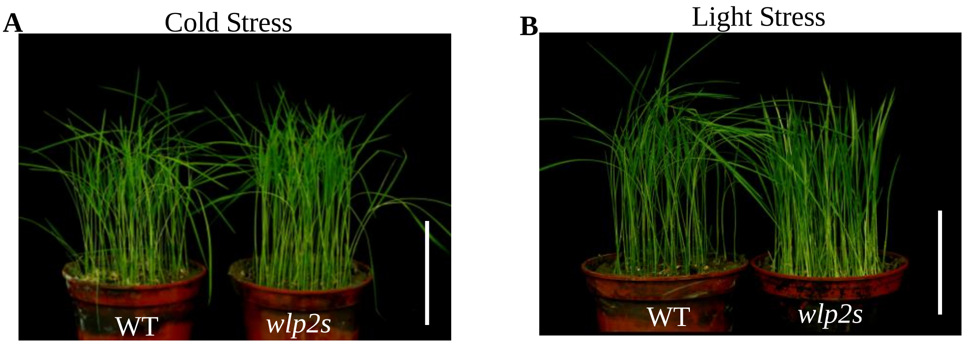

Fig. S10. The responses of two mutants and wild-type plants with other abiotic stresses. (A)The plants were grown under continuous 21 ℃ tmperature conditions in a growth chamber (12/12 h light/dark; light intensity 300 μmol m^{-2} s^{-1}). (B) Both the mutants and wild-type plants were germinated and cultivated under high light conditions with light intensity 1000 μmol m^{-2} s^{-1} (12/12 h light/dark, 26 ℃ temperature). Bars = 2 cm in A and B.

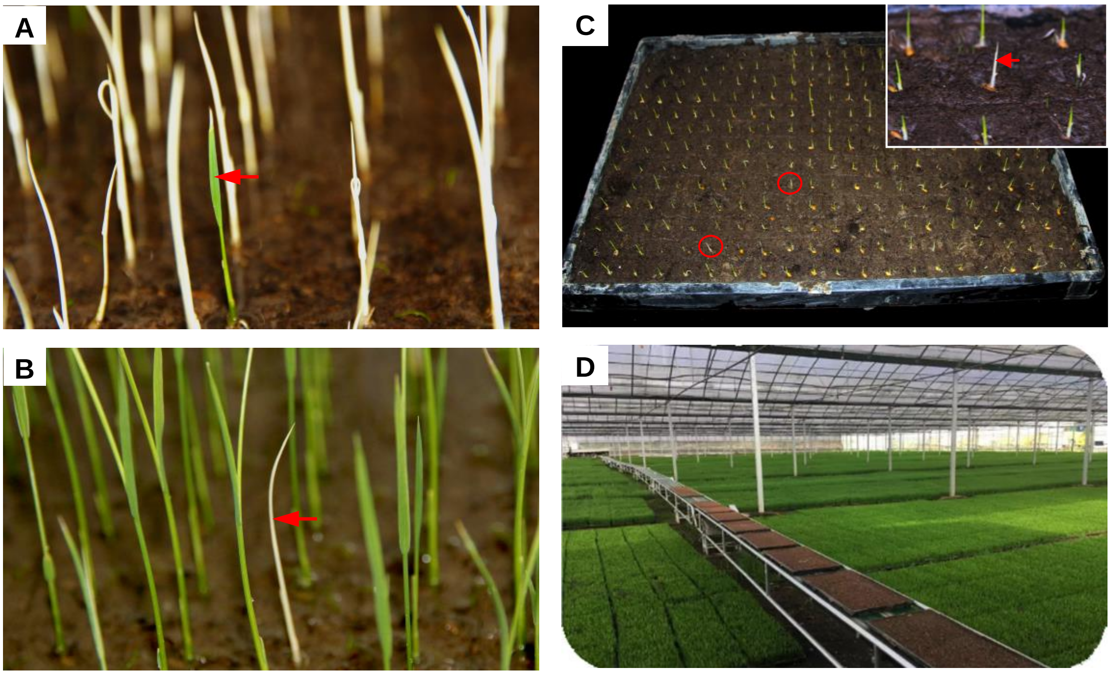

Fig. S11. Potential use of *wlp2w* as an early selective marker for enhancing seed purity and automated production of hybrid rice.

(A) The *wlp2w* acts as an early marker for eliminating the false seedlings to enhance sterility line purity. (B) Hybrids seeds derived from the cross MS Yu01s^{wlp2w} and the restorer line Huazhan were germinated and grown in an incubator at 32 ℃ continuous temperature. The green seedlings are true F_1 hybrids and the albino dead seedlings are off-type seeds (false hybrids) that derived from self-pollination of MS Yu01s^{wlp2w} (C) The *wlp2w* marker identifies self-pollination of the restorer lines during high through put production of hybrid seeds in trays at 32 ℃ constant temperature. (D) View of high through put industrial facility for the potential use of the *wlp2w* gene in the production of hybrid seeds.

Supplementary Table S1. Primer sequences used in this study.

Marker	Forward sequence (5'-3')	Reverse sequence (5'-3')
**For fine mapping**		
YS1	GCTGGTTGATTCAGCTAGTC	GCCTCGTTGTCGTTCCACAC
YS2	CACATGCTCTGGACACCAAC	GGAGCAAATAAGCCAACCAA
YS3	TAGATCTGGATGCCTCATGC	TTTACAAGCGTCCTCCTCCT
YS4	AGCATTAGGAAGGCACCAAA	CTCTGGCCAACATTGGTCTT
YS5	GGCCCACGTAATCTGATTTG	CGACGATCGGACTTATCAGC
YS6	GGGATTATTTGAAATCTTTGC	ATATAGCATTGCCAGTTTGC
YS7	GCCTGTAGTACCTGGCCTTG	CAGTCGCTGCTCCACGTAT
YS8	CCAAATTCTTTGTGCCCACT	GGTGCTGAAAATTGTGTTGC
YS9	TTGCCAGTTGAGCAACATCT	GGAAAGACTGCAATTTATTCTGAA
YS10	GCAGCCATGGTGTGTCTCT	GAGGAATGTCCTCTTTTTCTTGA
YS11	AATACTGTATCCTTGTGTAAGATTGC	CCCCTTAGCTAGAAGCCAGA
YS12	TGATTGATTGCTTGGCACTG	AGATAGCTGCCTGCATCG
YS13	TCCATCATCCATTCTGAGTTT	TTCTTCTCCGCTTCTGCTTC
Indel 10	TTGATAACAAGTTTGTTTTATGTTTTG	TTTGATCCCCAGATGAATGA
Indel 14	TGTTTTGGAAACAAGTAGTGATGT	CAGTGGATGTATGAACAAGTGGA
Indel 20	GCAGTTCCATGCTAGTATTTTCA	GCTGGAACACTCTTCCCAACT
**For quantitative PCR**		
PORA	TGTACTGGAGCTGGAACAACAA	GAGCACAGCAAAATCCTAGACG
rbcL	CTTGGCAGCATTCCGAGTAA	ACAACGGGCTCGATGTGATA
rbcS	TCCGCTGAGTTTTGGCTATTT	GGACTTGAGCCCTGGAAGG
psaA	GCGAGCAAATAAAACACCTTTC	GTACCAGCTTAACGTGGGGAG
psbA	CCCTCATTAGCAGATTCGTTTT	ATGATTGTATTCCAGGCAGAGC
WLP2	ACTCCCTCAATGGAAAGCACAC	CCTTGACCTCCACAAAGGCAAT
rpoA	GTGGAAGTGTGTTGAATCAA	TCTCTCTTGATCCGTAACTC
rpoB	TTTGGTTTCGATGTGCA	TATGGTCTAATTCCGAGCGGT
rpoTp	AAGCAGACAGTGATGACATC	ATCACATGCATGCACCCAAA
rps12	AGCCGTTTGCTACCAATGG	TGATCGGTACCAATGAATAGG
OsPPR1	CTAAGACCGAATGACAAATGC	GCACTGCCAACAAGAATACC
Oscab1R	AGATGGGTTTAGTGCGACGAG	TTTGGGATCGAGGGAGTATTT
Oscab2R	TGTTCTCCATGTTCGGCTTCT	GCTACGGTCCCCACTTCACT
CAO1	GATCCATACCCGATCGACAT	CGAGAGACATCCGGTAGAGC
YGL1	AACCTTACCGTCCTATTCCTT	CCATACATCTAACAGAGCACCC
V1(NUS1)	TGGAGGTCGGGACAGAGGA	CGAGGAGCACCACCATCAC
V2	CGACAAGCAGAGCGAAGCG	AGGTTGCTGCTCCTTGAATGT
APX1	TCCACCCAGGAAGGGAGG	TTGGTAGCATCAGGAAGACGG
APX2	TCCCCTACCCTGCTGCATC	ACCAGCCAACCACTCGCA

APX3	AGCACTCTCAGGCGGCC	AGGCACCATCAAATCCTGATCT
APX7	TTCACGTTGGACGGTTAATGC	TTTCTGTAAAAGTGGTTGGCCA
APX8	ATCATCGCCAGCGGATGA	GCAGCGACGAAGGGCTC
OsFLN2	AGGAGCCATTTACATTATAAGCC	AACTTACGTTTCGGTTGAGCA
OsTRXz	GCCTCCCTCTCCTGCGATG	CATTCCGTCGAACGCCTTG

For vector construction

1305ubi:WLP2-GFP	CaaggtaccTCCCACCGAAGGAGAGAGCC	CTTactagtACCAGTCCGCCAGTCACCAC
1381Z-GUS	CCGGAATTC CTAGCCCCCACAGTGAACATG	CCCAAGCTT GTTCGGGGAGCAGCGAATAT
p35S-WLP2-GFP	GCCCAGATCAACTAGTATGGCCATGGCGGCCTCCCC	TTTACTTTACTCTAGACCACATAGAAGGCACATATACTTGC
p35S-WLP2$^{1-250AA}$-GFP	CGGAGCTAGCTCTAGACGTCCCGACGGTGCGGGTGTCGGAC	TGCTCACCATGGATCCCCACATAGAAGGACATATACTTG
p35S-WLP2$^{1-150AA}$-GFP	CGGAGCTAGCTCTAGAATGGCCATGGCGGCCTCCCCATT	TGCTCACCATGGATCCCGGCGAATCCTCCCCGTCTTC
p35S-WLP2^{1-70AA}-GFP	CGGAGCTAGCTCTAGAATGGCCATGGCGGCCTCCCCATT	TGCTCACCATGGATCCCGTGGTCTTCGCCTTCG
p35S-WLP2^{1-50AA}-GFP	CGGAGCTAGCTCTAGAAAGAAGAATACGCAGGAGTCT	TGCTCACCATGGATCCGGGTTCAGGGGATTCCGGTAC
p35S-WLP2^{1-30AA}-GFP	CGGAGCTAGCTCTAGAATGGCCATGGCGGCCTCCCCATT	TGCTCACCATGGATCCGAAGATGCTGGGGTGGATGC
p35S-WLP2$^{50-531AA}$-GFP	CGGAGCTAGCTCTAGAGTACCGGAATCCCCTGAACCC	TTTACTTTACTCTAGACCACATAGAAGGCACATATACTTGC
pGADT7- TRXz	GGAGGCCAGTGAATTCATGGCCATGGCCGCGGCCGCCTC	CGAGCTCGATGGATCCACAATTCATTATCAATGATATTTCTGA
pGADT7- OsFLN2	GGAGGCCAGTGAATTC ATGCACCGAATGGCTTCTCTTCTTCTC	CGAGCTCGATGGATCC ACTCCACATATAAAAGCTCACTCTCT
pGADT7-WLP2	GGAGGCCAGTGAATTCATGGCCATGGCGGCCTCCCCATTCCT	CGAGCTCGATGGATCC ACCACATAGAAGGCACATATACTTGC
pGBKT7-WLP2	CATGGAGGCCGAATTCATGCACCGAATGGCTTCTCTTCTTCTC	TAGTTATGCGGCCGCTGCAGACTCCACATATAAAAGCTCACTCTCT
pGBKT7- TRXz	CATGGAGGCCGAATTCATGGCCATGGCCGCGGCCGCCTC	TAGTTATGCGGCCGCTGCAGACAATTCATTATCAATGATATTTCTGA
pGBKT7-pfkB	CATGGAGGCCGAATTCGACATATACTCCACGTGGCTGCATCT	TAGTTATGCGGCCGCTGCAGCTCAGTAGGGAATCCTCGCACAGCA
pSPYNE-OsFLN2	CGCCACTAGTGGATCC ATGCACCGAATGGCTTCTCTTCTTCTC	TACCCTCGAGGTCGAC ACTCCACATATAAAAGCTCACTCTCT
pSPYNE-WLP2	CGCCACTAGTGGATCCATGGCCATGGCGGCCTCCCCATTCCT	TACCCTCGAGGTCGACCCACATAGAAGGCACATATACTTGC
pSPYCE- TRXz	CGCCACTAGTGGATCCATGGCCATGGCCGCGGCCGC	TACCCTCGAGGTCGACCAATTCATTATCAATGATA

	CTC	TTTC
PGEX-4T-TRXz	GGTTCCGCGTGGATCCATGGCCATGGCCGCGGC	GTCGACCCGGGAATTCTCACAATTCATTATCAATGATA
pET-28a-OsFLN2	AATGGGTCGCGGATCCATGCACCGAATGGCTTC	GGTGGTGGTGCTCGAGTCACTCCACATATAAAAGCT
pfast-bacI-WLP2	CCACCATCGGGCGCGGATCCGCCACCATGCTGCTGGT	TAGTACTTCTCGACAAGCTTTCACCACATAGAAGGCACATATAC

Supplementary Table S2. Main agronomic traits of wild-type, *wlp2s* and *wlp2w* plants grown at the Hangzhou paddy field in 2014 and 2015.

**Year**	**Materia**	**Plant height (cm)**	**No. of tillers**	**Length of panicle (cm)**	**Seed setting rate (%)**	**1000-grain weight (g)**
2015	WT	97.88±2.90	11.33±2.08	22.73±1.20	0.57±0.01	24.15±0.19
	wlp2s	78.67±2.52*	12.67±1.53	21.57±0.97	0.19±0.05**	17.95±0.84**
	wlp2w	99.22±3.47	11.23±2.11	23.79±1.36	0.44±0.03*	21.06±0.23**
2014	WT	103.4±2.01	8.6±1.95	22.98±0.99	0.75±0.06	27.29±0.74
	wlp2s	90.78±1.49**	8.6±1.34	23.48±0.84	0.72±0.04	23.15±0.34**
	wlp2w	106.24±3.01	9.00±1.88	23.18±0.98	0.76±0.08	26.38 ±0.65

The mean temperature at the heading stage in the summer of 2014 and 2015 were 28.5 ℃ and 32.8 ℃, respectively. Data are shown as means ± SD from five replicates. The asterisks indicate statistical significance between the wild-type and the mutant, as determined by the Student's *t*–test (* $P < 0.05$; ** $P < 0.01$).

Supplementary Table S3. Genetic analysis for the *wlp2s* mutant gene.

The segregation behavior in each of the two derived F_2 populations was consistent with the Mendelian monogenic ratio of three wild type to one albino

**Cross**	**Number of wild type plants**	**Number of *wlp2s* type plants**	**χ^2（3:1）**	**P**
wlp2s/NJ11	5037	1607	2.340759	0.126
wlp2s/Peiai64	322	117	0.638573	0.424
